# Tumor microenvironment responsive nano-immunoregulator for precision cancer photodynamic immunotherapy

**DOI:** 10.1016/j.mtbio.2026.103162

**Published:** 2026-04-27

**Authors:** Xiaowei Chang, Miao Yu, Pan Wei, Jie Cheng, Yaping Wu

**Affiliations:** a Yan'an Medical College, Yan'an University, Yan'an, 716000, Shaanxi, China; b State Key Laboratory Cultivation Base of Research, Prevention and Treatment for Oral Diseases, Jiangsu Province Engineering Research Center of Stomatological Translational Medicine, Department of Oral and Maxillofacial Surgery, The Affiliated Stomatological Hospital, Nanjing Medical University, Nanjing, 210029, Jiangsu, China

**Keywords:** Tumor microenvironment responsive, Photodynamic immunotherapy, Precision nanomedicines, Primary tumor clearance, Metastasis inhibition

## Abstract

Spatiotemporally controlled reactive oxygen species (ROS) generation remains pivotal for advancing photodynamic immunotherapy. Herein, we present tumor microenvironment (TME)-responsive nano-immunoregulator featuring a CaCO_3_ shell encapsulating a mesoporous silica core modified with folic acid/chlorin e6 (FA/Ce6) and co-loaded with dBET6 and maleimide (MA). Acidic TME triggers CaCO_3_ shell degradation, neutralizing tumor acidity to repolarize macrophages toward M1 type, enhancing antigen presentation and T cells immune response while exposing the BM@M_FC_ core. The resulting BM@M_FC_ further endocytosed by tumor cells via FA-mediated tumor targeting, followed by MA-mediated glutathione depletion amplifies Ce6-generated ROS for potent photodynamic therapy (PDT). Concurrently, dBET6 degrades BRD4 to inhibit metastasis, downregulate PD-L1, and synergize with PDT to induce immunogenic cell apoptosis, thereby activating dendritic cells (DCs) and T cells for enhanced photodynamic immunotherapy. Both *in vitro*/*vivo* results indicate that BM@M_FC_C have excellent performance to inhibit primary/metastatic tumors, while single-cell RNA sequencing elucidates BM@M_FC_C can induce immunosuppressive TME remodeling, including increased CD8^+^ T cells, cDCs, and NK cells; M2-to-M1 macrophage polarization; and enhanced intercellular communication. Tumor cell profiling confirms BM@M_FC_C will downregulate oncogenic pathways and activate immune signatures. Collectively, this TME responsive nano-immunoregulator demonstrates superior therapeutic outcomes and provides a promising strategy for precision cancer photodynamic immunotherapy.

## Graphical abstract

**Figure:**
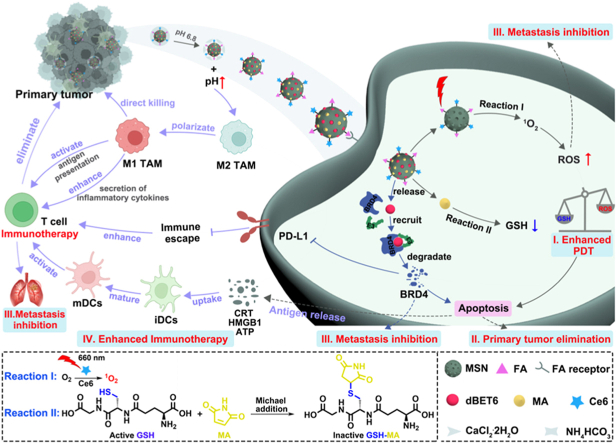

## 1 Introduction

Oral squamous cell carcinoma (OSCC), the predominant histological subtype of oral malignancies, originates primarily from the malignant transformation of mucosal epithelium in multiple anatomical sites including the tongue, buccal mucosa, floor of the mouth, and alveolar ridges. Accounting for over 90% of all oral cavity cancers according to WHO classification, this aggressive epithelial neoplasm poses significant clinical challenges and socioeconomic burdens globally [1]. Despite the notable anti-tumor efficacy of traditional therapies (surgery, chemotherapy, and radiotherapy) in OSCC treatment, effective metastasis inhibition and recurrence control remain challenging. This is attributed to the high tumor heterogeneity and strong invasive potential in OSCC, which collectively contribute to suboptimal long-term clinical outcomes for affected patients [2]. Tumor immunotherapy, a revolutionary approach that activates patients’ innate immune systems to target malignant cells, has emerged as a promising therapeutic strategy for OSCC [3]. However, current immunotherapeutic regimens demonstrate limited clinical efficacy in OSCC patients, underscoring the urgent need for developing next-generation approaches to enhance treatment efficacy.

Photodynamic therapy (PDT) damages primary tumor cells via photosensitizers transform oxygen to cytotoxic ROS under laser irradiation, has received widespread attention and approved by the Food and Drug Administration for treatment of various tumors [4]. Recent studies also identified that PDT can *in situ* generate tumor-associated antigens (TAAs) during damaging tumor cells, which further taken up by immature dendritic cells (iDCs) to promote their maturation, subsequently delivered the tumor antigen to T cells for activating immunotherapy [5]. These insights have inspired that the manufacture of nano-immunoregulator using photosensitizer to produce tumor antigens as needed *in vivo*, hopefully overcome patient heterogeneity and improve treatment effect of tumors. However, the overexpressed glutathione (GSH, major reducing substances) in tumor cells probably reduce the therapeutic efficacy of this photodynamic immunotherapy via directly eliminating ROS [6]. Moreover, conventional photosensitizers may randomly endocytose in the surrounding immune cells to damage these cells through non-selective ROS generation, reducing the efficiency of immunotherapy [7]. Therefore, nanoparticles capable of the precise and effective ROS production in tumor cells are ideal nano-immunoregulator candidates for photodynamic immunotherapy.

Despite the tumor-specific photodynamic nanoparticles have the potentiality as a new type immunotherapy strategy, the immune response caused by TAAs only may not be enough to realize the treatment of malignant tumors, due to the immunosuppressive TME remarkably weakening the anti-tumor immune response [8]. Generally, the immunosuppressive TME is closely related to the type of macrophages, which are divided into M2 type (promoting tumor cell growth) and M1 type (suppressing tumor growth) [9]. However, the tumor tissue mainly is M2 type. In addition, the overexpressed bromodomain protein 4 (BRD4) in tumor cells promotes the proliferation and metastasis, as well as upregulates PD-L1 expression to promote immune escape [10]. Therefore, the therapeutic nanoparticles can induce photodynamic immunotherapy via precise and efficient ROS generation, meanwhile reverse the immunosuppressive TME, will be a potent strategy for treatment of OSCC.

**Scheme 1:**
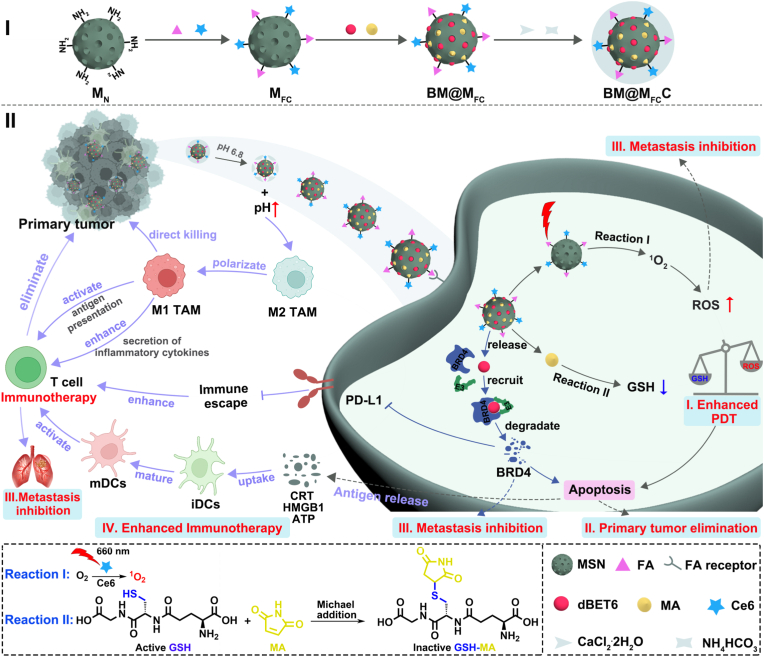
**Schematic illustration of fabrication (I) and therapeutic mechanism (II) of BM@M_FC_C nano-immunoregulator.** The BM@M_FC_C are able to efficiently and precisely generate ROS in tumor to activate PDT and inhibit tumor metastasis, which further induce apoptosis for primary tumor elimination and produce TAAs for activating anti-tumor photodynamic immunotherapy. In addition, the dBET6 induces BRD4 degradation synergistically enhance apoptosis, metastasis inhibition, and reduce PD-L1 expression. Meanwhile, the local pH increase in tumor tissue promotes M2-to-M1 macrophage polarization, causing additional direct tumor cell killing, activating antigen-presenting immune responses, and enhancing anti-tumor immunity through inflammatory cytokine secretion.

Herein, utilizing tumor specific acidic microenvironment, we fabricated a pH-responsive nano-immunoregulator (dBET6/MA@MSN-FA/Ce6-CaCO_3_, BM@M_FC_C) to perform on-demand photodynamic immunotherapy while regulate the immunosuppressive TME. In preparation, FA (tumor targeting agent) [11] and Ce6 (photosensitizer) [12] were first coupling on the mesoporous silica nanoparticles (MSNs), followed by co-loading of dBET6 (BRD4 PROTAC) [13] and MA (GSH depleting agent) [14], and further coated with CaCO_3_ shell to obtain the final BM@M_FC_C. After application, the acidic TME first degraded CaCO_3_ shell [15], subsequently exposed BM@M_FC_ core and raised the pH of tumor tissue to polarize macrophages, enhance antigen presentation. The resulting BM@M_FC_ further taken up by tumor cells through FA-induced tumor targeting, and exhibited the following three-step chain reaction: 1) the covalent linkage of Ce6 convert endogenous oxygen into cytotoxic ROS for PDT and further inducing immunogenic cell apoptosis; 2) release of MA strengthen PDT via depleting GSH; 3) release of dBET6 degrade BRD4 to coordinatively induced apoptosis, inhibited metastasis and PD-L1 expression. The apoptosis caused by PDT and BRD4 degradation synergistically generated abundant TAAs (CRT, HMGB1 and ATP) to mature iDCs and activate T cells for enhanced photodynamic immunotherapy (Scheme 1). Both *in vitro*/*vivo* results indicated that BM@M_FC_C had excellent performance to inhibit the primary and metastasis tumor. Single-cell RNA sequencing of SCC7 tumor tissues treated with BM@M_FC_C revealed enhanced anti-tumor immunity with increased CD8^+^ T cells, cDCs, macrophages, and NK cells, and decreased tumor cells. Macrophage polarization analysis showed a 2.89-fold increase in M1-polarized macrophages, indicating successful macrophage repolarization. CellChat analysis demonstrated enhanced immune coordination, with strengthened interactions between M1-like macrophages and CD8^+^ T cells, and intensified cDC-CD8^+^ T cell crosstalk. Malignant epithelial cell analysis showed BM@M_FC_C inhibit tumors via modulating oncogenic pathways and activating tumor-intrinsic immune signals, such as oxidative stress response, EMT inhibition, PD-L1 downregulation, and immune activation pathways. Therefore, our BM@M_FC_C could be a potent candidate for photodynamic immunotherapy to treatment of malignant tumors.

## 2 Results and discussion

### 2.1 Fabrication and characterization of BM@M_FC_C

In order to prepare the BM@M_FC_C, MSNs were first synthesized according to our laboratory's previous studies [16], and then reacted with 3-(triethoxysilyl)propan-1-amine (amino silane coupling agent) to synthesize amino modified MSNs (MSN-NH_2_, M_N_). Then, the FA and Ce6 molecule were modified on M_N_ through amidation reaction between -NH_2_ and -COOH to obtain MSN-FA/Ce6 (M_FC_). After that, the as-prepared M_FC_ were loaded with dBET6 and MA to form dBET6/MA@MSN-FA/Ce6 (BM@M_FC_), which were further sedimented a CaCO_3_ shell via gas diffusion method to obtain the final dBET6/MA@MSN-FA/Ce6-CaCO_3_ (BM@M_FC_C) nano-immunoregulator. Considering the FA, Ce6, dBET6 and MA have independent functions without competition and the therapeutic effect of these drugs are dose-dependent, therefore the excessive agents were employed to saturate each component, ensuring optimal performance. The exact mass ratio of each component in BM@M_FC_C were determined by thermogravimetric analysis of M_N_, M_F_, M_FC_, B@M_FC_ and BM@M_FC_, which indicated that weight percentage of FA, Ce6, dBET6 and MA were 11.4%, 11.9%, 12.8% and 8.8%, respectively (Fig. S1).

**Fig. 1:**
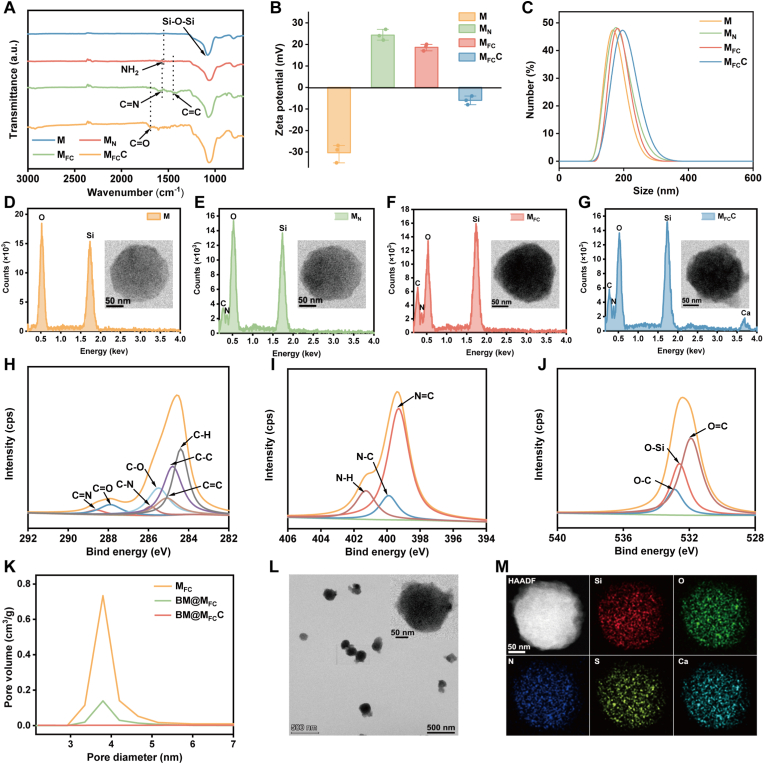
**Fabrication and characterization of BM@M_FC_C**. A) FTIR spectra, B) Zeta potential and C) DLS results of M, M_N_, M_FC_ and M_FC_C. D-G) EDX spectroscopy and TEM image of M, M_N_, M_FC_ and M_FC_C. H-J) Deconvoluted high resolution XPS spectra of C 1s, N 1s, and O 1s regions of M_FC_C, confirming the structure of M_FC_C skeleton. K) Pore size distribution of M_FC_, BM@M_FC_ and BM@M_FC_C, confirming the dBET6 and MA successfully co-loaded into pores of M_FC_. L) TEM imaging of BM@M_FC_C. M) Elemental mapping analysis of BM@M_FC_C. M represents MSNs; M_N_ represents amino group modified MSNs; M_FC_ represents FA and Ce6 modified MSNs; M_FC_C represents M_FC_ with CaCO_3_ shell; BM@M_FC_ represents dBET6 and MA co-loaded M_FC_; BM@M_FC_C represents BM@M_FC_ with CaCO_3_ shell.

The stepwise fabrication of M_FC_C nanocarrier without dBET6 and MA loading was first investigated by fourier-transform infrared spectroscopy (FTIR), which gradually appeared characteristic stretch vibration of Si-O-Si from MSNs, -NH_2_ from amino silane coupling agent, C=N from FA, C=C from Ce6, along with C=O from CaCO_3_ shell (Fig. 1A). Further characterization was achieved through zeta potential and dynamic light scattering (DLS). As indicated in Fig. 1B and C**,** the expected change of surface electric charge in line with the electronic properties of surface components, as well as a slightly increase of the nanoparticle's hydration kinetics diameter after each step.

The chemical element and microstructure of as-prepared M_FC_C skeleton and various intermediate products were studied by energy dispersive spectrometer (EDS) and transmission electron microscopy (TEM). Although the M, M_N_, M_FC_, M_FC_C all depicted a spherical morphology with diameter of 160 nm to 200 nm, the typical elements such as Si (from MSNs), N (from amino silane coupling agent, FA and Ce6) and Ca (from CaCO_3_ shell) only appeared in the specific nanoparticles in accordance with decoration steps (Fig. 1D–1G). The chemical constitution of M_FC_C were further evaluated by X-ray photoelectron spectroscopy (XPS), which presented characteristic peaks of C 1s (C=N, C-N) and N 1s (N=C, N-C) from FA and Ce6, O 1s (O-Si) from MSNs, as well as C 1s (C=O, C-O) from CaCO_3_ shell (Fig. 1H–1J). All these results suggested that the successful fabrication of M_FC_C nano-skeleton.

The drugs loading procedure of dBET6 and MA to obtain final BM@M_FC_C were first studied by N_2_ absorption isotherm experiments, which obviously observed that dBET6 and MA co-loading leaded to clearly drop of pore volume, and pores were completely blocked by deposition of CaCO_3_ shell (Fig. 1K). These findings proved that the successful loading of dBET6 and MA, and CaCO_3_ shell could be used as a sealing agent to prevent early release of encapsulated drugs until the BM@M_FC_C arrived to the acidic tumor tissue. The maximum loading content and encapsulation efficiency were 12.3% and 86.3% for dBET6, and 8.2% and 82.3% for MA, respectively. The structural characterization of the BM@M_FC_C were provided by IR, XPS, UV-Vis spectroscopy, Zeta potential and DLS. The FTIR spectrum of BM@M_FC_C displayed characteristic peaks corresponding to Si-O-Si from MSNs, C=N from FA, C=C from Ce6, along with C=O from CaCO_3_ shell (Fig. S2A). The XPS spectra showed typical peaks for all expected elements, including Si (2p), S (2p), O (1s), N (1s), Ca (2p) and C (1s) (Fig. S2B). UV-Vis spectroscopy exhibited the absorption peak of dBET6 at 329 nm and MA at 272 nm, collectively indicating successful drug loading (Fig. S2C). The surface charge of BM@M_FC_C was −14.6 mV, which is consistent with the charge of the surface modification molecules (Fig. S2D). The hydrodynamic diameter and PDI of BM@M_FC_C were 196 nm and 0.21, confirming the nanoscale size and narrow distribution (Fig. S2E). More information of BM@M_FC_C were provided by TEM and mapping model of EDS (Fig. 1L and M), which clearly depicted that BM@M_FC_C had a monodisperse spherical structure with a diameter of approximately 195 nm, along with the characteristic element of Si, O, N, S and Ca all homogeneously distributed and fully filled the outline of MSNs. All these results indicated that the successful construction of BM@M_FC_C nano-immunoregulator.

### 2.2 pH-triggered drugs release, function and stability of BM@M_FC_C

**Fig. 2:**
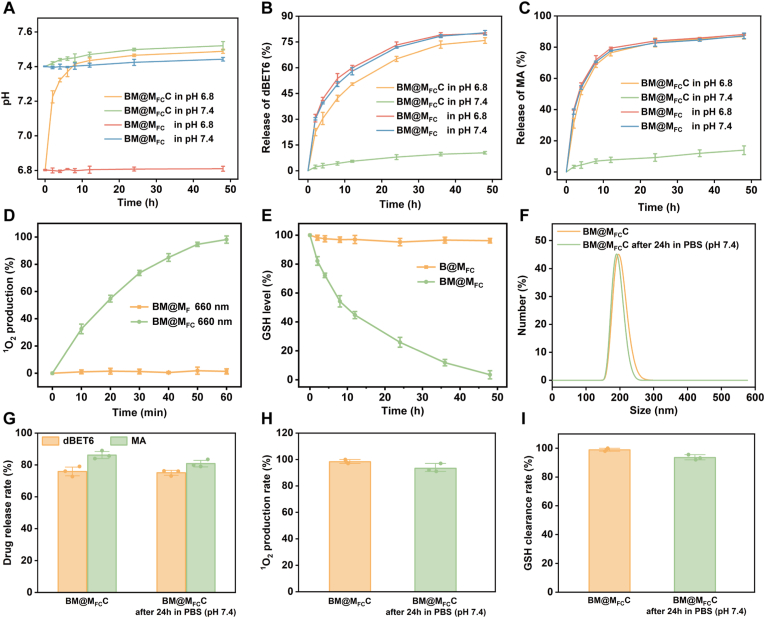
**pH-triggered drugs release, function and stability of BM@M_FC_C**. A) pH changes of BM@M_FC_ and BM@M_FC_C at pH 6.8 and pH 7.4. pH-responsive release profiles of dBET6 (B) and MA (C) from BM@M_FC_C in buffers at different pH values. D) ^1^O_2_ production rate of BM@M_F_ and BM@M_FC_ under 660 nm irradiation, E) GSH level after incubation with B@M_FC_ and BM@M_FC_, which was investigated by Ellman's assay. F) DLS of BM@M_FC_C before and after 48 h incubation at pH 7.4, confirming structural stability of BM@M_FC_C. G) Release of dBET6 and MA, H) ^1^O_2_ generation and I) GSH clearance of BM@M_FC_C, which with or without incubated in PBS (pH 7.4) for 24 h, confirming the functional stability of BM@M_FC_C. B@M_FC_ represents dBET6 loaded M_FC_s; BM@M_FC_ represents dBET6 and MA co-loaded M_FC_.

According to design, BM@M_FC_C decomposed CaCO_3_ shell at specific acidic TME to raise local pH and disclosed BM@M_FC_ core. Therefore, both BM@M_FC_ and BM@M_FC_C were incubated in PBS at pH 6.8 (mimicking acidic TME) and 7.4 (mimicking normal physiological microenvironment), and the pH was measured after at predetermined time points. As shown in Fig. 2A, BM@M_FC_C significantly raised the pH from 6.8 to 7.4, whereas BM@M_FC_ caused no change, and at pH 7.4, BM@M_FC_C had little effect on pH while BM@M_FC_ remained ineffective. After 48 h incubation at pH 6.8, BM@M_FC_C showed a significant size decreased, due to CaCO_3_ shell dissolution, whereas the BM@M_FC_ maintained a stable size (Fig. S3A). In addition, zeta potential of BM@M_FC_C changed dramatically from −14.3 mV (pH 7.4) to 11.6 mV (pH 6.8), consistent with the loss of CaCO_3_ shell and exposure of BM@M_FC_ core (Fig. S3B). Furthermore, we collected the BM@M_FC_C nanoparticles, which have incubated at pH 6.8 for 48 h, and examined by TEM-EDS mapping. The Ca signal completely disappeared after acidic incubation (Fig. S3C), directly demonstrating the dissolution of CaCO_3_ shell to exposure BM@M_FC_. Subsequently, pH-dependent drug release profiles of dBET6 and MA from BM@M_FC_ and BM@M_FC_C were systematically evaluated under pH 6.8 and pH 7.4. As can be see from Fig. 2B and C, BM@M_FC_C demonstrated a clear pH-dependent release pattern for both drugs, with 76 % ± 2 % dBET6 and 86 % ± 2 % MA at pH = 6.8, whereas only 10 % ± 1 % dBET6 and 14 % ± 3 % MA was released at pH = 7.4 for 48 h. In contrast, BM@M_FC_ exhibited rapid and sustained release irrespective of pH, confirming the absence of a gating mechanism (CaCO_3_ shell). Collectively, these results unequivocally characterized the pH-sensitivity of the BM@M_FC_C and confirm that the pH-responsive behavior is indeed attributable to the CaCO_3_ shell.

To further characterize the PDT functionality, we quantitatively assessed the Ce6-mediated ROS generation capacity of BM@M_FC_ using 1,3-diphenylisobenzofuran (DPBF) as a ROS-sensitive spectrophotometric probe under 660 nm irradiation [17]. As can be seen from Fig. 2D, the BM@M_FC_ significantly generated ROS (^1^O_2_) under 660 nm irradiation, however BM@M_F_ without Ce6 modification basically not produced ROS, indicating that BM@M_FC_ could effectively produce ROS for PDT. Considering strong reactivity of MA with thiols via Michael addition, the BM@M_FC_ were expected to combine with overexpressed GSH in tumor cell to inactivate GSH, hopefully reducing the free radical scavenging ability of tumor cells to enhance PDT. Therefore, the GSH inactivation property of BM@M_FC_ was studied by Ellman's assay [18]. As evidenced in Fig. 2E, BM@M_FC_ demonstrated significant GSH depletion with time-dependent kinetics, in stark contrast to the B@M_FC_ control group (MA-deficient formulation) where intracellular GSH levels remained stable, demonstrating that BM@M_FC_ could effectively reduce GSH levels that overexpressed in tumor cell for enhancing PDT.

In nanomaterial-based tumor therapy, the storage, structure and function stability is essential for assessing their practical applicability and *in vivo* behavior. Therefore, the BM@M_FC_C were first stored at 4 °C (sealed, protected from light) for 30 days to evaluate the storage stability. At predetermined time points (0, 3, 7, 14, 30 days), we monitored the hydrodynamic size, PDI, zeta potential, and drug loading efficiency. The results showed that BM@M_FC_C maintained stable particle size (195 nm ± 5 nm) with PDI <0.25 (Fig. S4A), unchanged zeta potential (Fig. S4B), and drug loading efficiency of dBET6 and MA remained about12.3% and 8.2%, respectively (Fig. S4C). These data confirmed that BM@M_FC_C nanoparticles have good storage stability for at least 30 days at 4 °C. In addition, the structure and function stability of BM@M_FC_C after 24 h incubation in PBS (pH 7.4) and 10% fetal bovine serum (FBS) was investigated to verify the systematic stability in biosystem. The hydration kinetic diameter of BM@M_FC_C before and after 24 h incubation in PBS and FBS was first studied by DLS. The average size of BM@M_FC_C had no significant change after incubation in PBS or FBS for 24 h (Fig. 2F and S5A), demonstrating the high structure stability of BM@M_FC_C. After that, the pH-responsive release kinetics of dBET6 and MA from BM@M_FC_C, which had incubated in PBS or FBS for 24 h, were systematically analyzed via real-time fluorescence spectroscopy under simulated acidic TME conditions (PBS pH 6.8). The payloads still exhibited sustained release profiles with cumulative liberation rates of dBET6 and MA both >75% within 48 h (Fig. 2G and S5B). As expected, the BM@M_FC_ also produced ROS under 660 nm irradiation (Fig. 2H and S5C), as well as depleted GSH (Fig. 2I and S5D). All these results indicated that BM@M_FC_C was highly stable when used *in vivo*.

### 2.3 *In vitro* anti-tumor performance of BM@M_FC_C

**Fig. 3:**
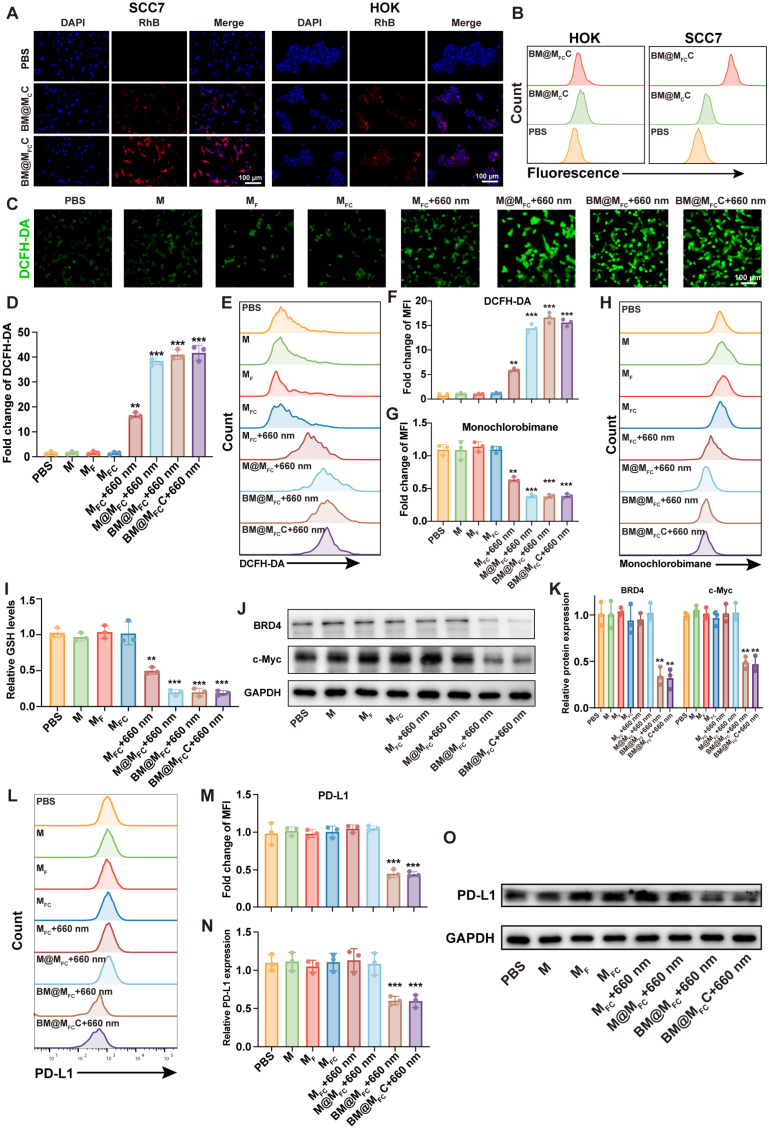
***In vitro* anti-tumor properties of BM@M_FC_C**. A) Confocal fluorescent microscope images of SCC7 cells and HOK cells after 24 h incubation with rhodamine B (RhB)-labeled BM@M_C_C and BM@M_FC_C, in which nucleus was pre-labeled by DAPI fluorescence dye. B) Flow cytometry analysis of HOK cells and SCC7 cells after 24 h incubation with RhB-labeled BM@M_C_C, BM@M_FC_C or PBS. C, D) Fluorescent microscope images and statistics of SCC7 cells after DCFH-DA staining and 48 h treatment of various formulas qualitatively assessed the intracellular ROS generation. The scale bar is 100 μm. E, F) Flow cytometry analysis and statistics of SCC7 cells after DCFH-DA staining quantitatively assessed the intracellular ROS generation. G-I) Intracellular GSH levels in SCC7 cells after various treatments. J, K) Intracellular BRD4 and c-Myc expression in SCC7 cells after various treatments, evaluated by western blotting analysis. The PD-L1 expression on SCC7 cells membrane after various treatments, assessed by flow cytometry (L, M) and western blotting (N, O). M represents MSNs; M_F_ represents FA modified MSNs; M_FC_ represents FA and Ce6 modified MSNs; M@M_FC_ represents M_FC_ after MA loading; BM@M_FC_ represents M_FC_ after dBET6 and MA co-loaded; BM@M_FC_C represents BM@M_FC_ with CaCO_3_ shell. Illumination (660 nm laser, 0.15 W, 5 min) was performed 4 h after adding nanoparticles to cells. Values are presented as mean ± SEM (n = 3). ∗P < 0.05, ∗∗P < 0.01 and ∗∗∗P < 0.001.

Prior to evaluating anti-tumor abilities of BM@M_FC_C, we first examined the expression of folate receptor FOLR1 on normal oral keratinocytes (HOK) and murine squamous cell carcinoma (SCC7) cells. The results showed that FOLR1 was significantly overexpressed in SCC7 cells compared to HOK cells (Fig. S6), confirming the selective overexpression of folate receptors on the tumor cell surface. Subsequently, cellular internalization of our nanoparticles was systematically investigated using confocal fluorescent microscope and flow cytometry. As illustrated in Fig. 3A, the red fluorescence from rhodamine B (RhB) labeled BM@M_FC_C in SCC7 cells was much stronger than that in HOK cells. In contrast, BM@M_C_C (FA-deficient nanoparticles), both HOK cells and SCC7 cells showed similarly negligible red fluorescence. Quantitative cellular uptake analysis was performed using flow cytometry, PBS group was included for comparison. As shown in Fig. 3B and S7, in HOK cells, both BM@M_C_C and BM@M_FC_C groups exhibited only slightly increased fluorescence compared to PBS group, with similar fluorescence intensity between the two treatment groups, indicating limited cellular uptake. In contrast, in SCC7 cells, the BM@M_FC_C group exhibited significantly higher fluorescence compared to both PBS and BM@M_C_C group, whereas both cell types co-incubated with BM@M_C_C displayed weak fluorescence. We further evaluated the time dependent cellular uptake of BM@_FC_C after co-culture with SCC7 cells. As can be seen from Fig. S8, a large number of the nanoparticles are internalized by cancer cells within 4 h. These results validated that BM@M_FC_C could achieve tumor-selective cellular entry through high-affinity binding to FA receptor overexpressed on SCC7 membranes, enabling precise co-delivery of Ce6, MA, and dBET6 for achieving synergistic anti-tumor therapy.

According to design, these internalized nanoparticles in tumor cells further delivery Ce6 to produce cytotoxic ROS (^1^O_2_) under 660 nm irradiation for photodynamic immunotherapy, release MA to deplete GSH for enhancing PDT, as well as release dBET6 to inhibit the expression of BRD4 for synergistically oncotherapy. Therefore, the ROS level in tumor cells after treatments of different nanoparticles was first investigated using 2′,7′-dichlorofluorescein diacetate (DCFH-DA) as a redox-sensitive fluorescent probe [19]. The probe will deacetylate by intracellular esterases to form DCFH, which is subsequently oxidized by ROS to yield fluorescent 2′,7′-dichlorofluorescein. The resulting green fluorescence was quantitatively analyzed using fluorescence microscopy, with signal intensity directly correlating to cellular ROS levels. The PBS (blank control), M (negative control), M_F_ (only targeting), M_FC_ (targeting + PDT unactuated), M_FC_ with 660 nm irradiation (targeting + PDT actuated), M@M_FC_ with 660 nm irradiation (targeting + PDT actuated + GSH depletion), BM@M_FC_ with 660 nm irradiation (targeting + PDT actuated + GSH depletion + BRD4 inhibition) and BM@M_FC_C with 660 nm irradiation (targeting + PDT actuated + GSH depletion + BRD4 inhibition + CaCO_3_ shell coating) were used as experimental group. As can be seen from Fig. 3C–3F, the intracellular ROS (green fluorescence) obviously increased in SCC7 cells after treatment by M_FC_ with 660 nm irradiation comparing with PBS, M, M_F_ and M_FC_ group. The ROS level further increased after treatment by M@M_FC_ with 660 nm irradiation, which basically unchanged by BM@M_FC_ and BM@M_FC_C both with 660 nm irradiation. These results demonstrated that the covalent connected Ce6 converted endogenous oxygen into cytotoxic ROS under 660 nm irradiation, the loaded MA depleted GSH to enhance ROS generation, and dBET6 loading and CaCO_3_ shell coating had little effect on ROS production. Therefore, BM@M_FC_C had great potential to efficiently generate ROS for photodynamic immunotherapy.

Considering MA could specifically couple with thiols of GSH via Michael addition in tumor cell [20]. Our nanoparticles expected to deplete the active thiols of GSH and lead to GSH invalidation for enhancing photodynamic immunotherapy. Therefore, the tumor intracellular GSH level after different treatments was also detected by Ellman's assay. As shown in Fig. 3G–3I, the GSH level of M, M_F_, M_FC_ treated group basically constant, similar to the PBS group. In contrast, the GSH level significantly decreased in SCC7 cells after treatment M_FC_ with 660 nm irradiation, due to Ce6 induced ROS generation consumed certain GSH. The GSH level further decreased after additional MA loading (M@M_FC_ with 660 nm irradiation group), which not further reduced by dBET6 loading (BM@M_FC_ group) and CaCO_3_ shell coating (BM@M_FC_C group). The GSH level in HOK cells showed only a modest decrease after MA-containing treatment compared to the PBS control, which was significantly less pronounced than that observed in SCC7 cells (Fig. S9). These results indicated that indicating our nanoparticles could reduce intracellular GSH level only in tumor cell for enhancing PDT and subsequent immune activation.

In addition, the overexpressed BRD4 in tumor functionally modulated three critical oncogenic pathways: anti-apoptosis, pro-metastasis, and upregulation of PD-L1 expression. Our nanoparticles hopefully released dBET6 to degrade the BRD4 protein, eliciting multimodal therapeutic effects including restored apoptotic sensitivity, inhibited metastasis, and downregulated PD-L1 expression. Therefore, the intracellular BRD4 and its downstream effector protein c-Myc expression after various treatments were measured by western blotting. As depicted in Fig. 3J and K, the intracellular BRD4 and c-Myc expression obviously decreased after treatment by BM@M_FC_ comparing with M, M_F_, M_FC_, and M@M_FC_ with or without 660 nm irradiation, while little affect after CaCO_3_ shell coating (BM@M_FC_C with 660 nm group), indicating BM@M_FC_C could effectively inhibit BRD4 and c-Myc expression for synergistic enhancement of tumor treatment. Furthermore, flow cytometry and western blotting analysis assessed PD-L1 protein expression across treatment groups. Notably, BM@M_FC_ and BM@M_FC_C exhibited the most pronounced suppression of PD-L1 (Fig. 3L–3O), mechanistically attributed to its capacity to degrade BRD4 and subsequently inhibit PD-L1 expression, indicating our nanoparticles could inhibit PD-L1 mediated immune escape to enhance photodynamic immunotherapy.

**Fig. 4:**
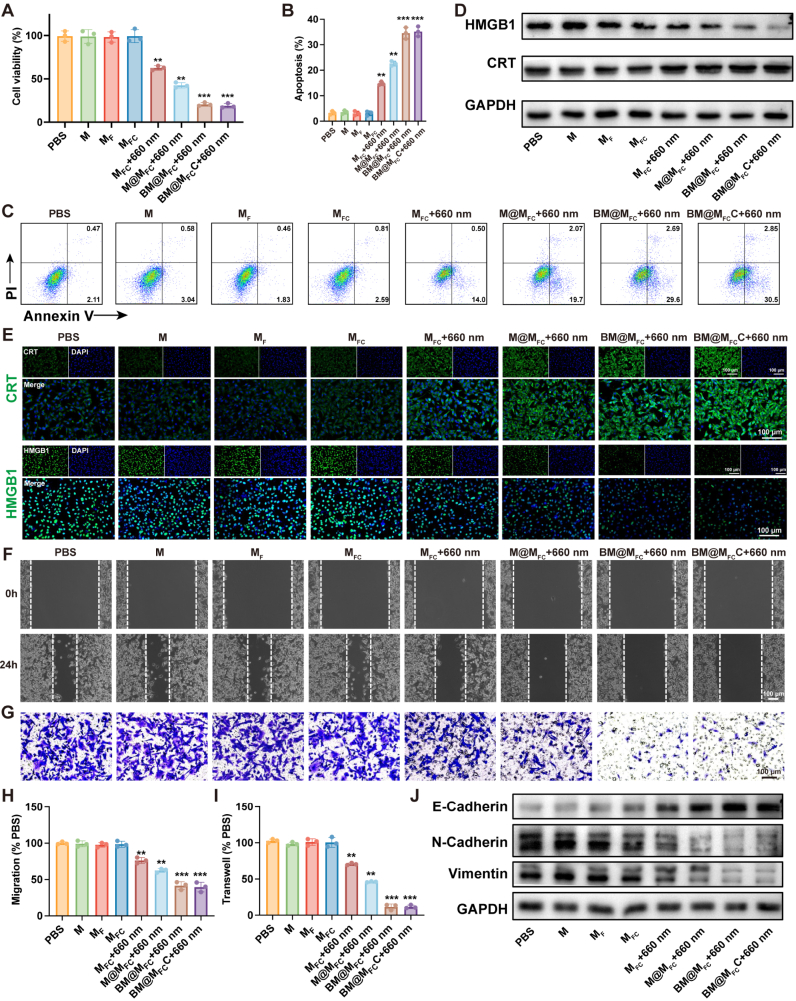
***In vitro* induce apoptosis and inhibit cell migration of BM@M_FC_C**. A) Cell viability of SCC7 cells after incubated with PBS, M, M_F_, M_FC_, M_FC_ + 660 nm irradiation, M@M_FC_ + 660 nm irradiation, BM@M_FC_ + 660 nm irradiation and BM@M_FCC_ + 660 nm irradiation. B, C) Apoptosis assay of SCC7 cells after different treatments. D, E) Western blotting analysis and fluorescent microscope images analysis of HMGB1 and CRT in SCC7 cells after various treatments. F, H) Wound healing assay of SCC7 cells in various groups. G, I) Transwell invasion assay of SCC7 cells in various groups. J) Migration relative protein expressions (E-cadherin, N-cadherin and Vimentin) in SCC7 cells after different treatments, evaluated by western blotting analysis. Values are presented as mean ± SEM (n = 3). ∗P < 0.05, ∗∗P < 0.01 and ∗∗∗P < 0.001.

The overall anti-tumor effect *in vitro* after various treatments was quantitatively assessed through CCK8 assays. As can be seen from Fig. 4A, the M_FC_ showed neglected cytotoxicity to SCC7 cells similar with PBS, M, M_F_ treated group, due to Ce6 induced PDT unactuated without 660 nm irradiation. Once the SCC7 cells was treated by M_FC_ with additional 660 nm irradiation, a lower cell viability at 62.67% was detected, which attributed to Ce6 catalyzed oxygen to produce ROS for activating PDT. The survival rate was further decreased to 42.67% after MA loading (M@M_FC_ with 660 nm irradiation) to deplete GSH for enhancing PDT. As expected, the lowest cell survival rate appeared in the BM@M_FC_ and BM@M_FC_C with 660 nm irradiation group, ended with less than 20.00% of surviving cells due to synergistic effect of tumor targeting, PDT activation, GSH depletion, and BRD4 protein degradation, demonstrating that BM@M_FC_C have a potential application for tumor therapy. To quantitatively evaluate the synergistic effect between dBET6 and Ce6, we performed combination analysis using the ZIP synergy model in SCC7 and B16F10 cells. The calculated ZIP synergy scores were 16.173 for SCC7 and 11.254 for B16F10 (Fig. S10), indicating a strong synergistic tumor-killing effect. Importantly, to evaluate the safety of these nanoparticles toward normal cells, we performed parallel cytotoxicity assays using HOK cells. As shown in Fig. S11, all nanoparticle formulations, including BM@M_FC_C with 660 nm irradiation, exhibited minimal inhibition of HOK cell proliferation, with cell viability remaining above 90%. This is consistent with the low expression of folate receptors on HOK cells, leading to reduced uptake of the folate-targeted nanoparticles and thus high biocompatibility.

The flow cytometric apoptosis analyses were well consistent with results of CCK8 assays. BM@M_FC_C with 660 nm irradiation group induced the highest apoptosis rate, compared with other groups, resulting from the combination of efficient PDT activation and BRD4 degradation (Fig. 4B and C). In addition to inducing tumor cell apoptosis, previous studies have indicated that PDT also activate anti-tumor immunity by causing immunogenic cell death (ICD). Mechanistically, PDT promotes the release of TAAs (CRT, HMGB1 and ATP), thereby triggering an adaptive immune response [21]. Therefore, the CRT and HMGB1 after PBS, M, M_F_, M_FC_, M@M_FC_, BM@M_FC_, or BM@M_FC_C treatment was investigated using western blotting and immunofluorescence staining analysis. Aligned with the degree of apoptosis, treatment with BM@M_FC_C with 660 nm irradiation resulted in a notable expression and relocation of CRT, as indicated by the obvious CRT protein band and green fluorescence signal originating from the plasma membrane. In addition, the HMGB1 protein was transported through nucleus region to the extracellular space (Fig. 4D and E and S12A, S12B). Furthermore, the ATP levels were gradually increased in M_FC_+660 nm, M@M_FC_+660 nm, BM@M_FC_+660 nm, and BM@M_FC_C+660 nm groups compared to control group, with the highest ATP concentration observed in the BM@M_FC_C+660 nm group, consistent with the changes in CRT and HMGB1 (Fig. S13). These results suggested that BM@M_FC_C can effectively induce apoptosis and ICD for activating photodynamic immunotherapy.

Given the significant role of ROS and BRD4 in tumor metastasis, BM@M_FC_C would inhibit metastasis of tumor cells. The anti-metastasis efficacy was assessed using wound-healing assays and transwell migration assay. As illustrated in Fig. 4F–4I, consistent with intracellular ROS levels and dBET6 protein expression, after treatments by M_FC_ and M@M_FC_ with 660 nm irradiation, the inhibition of migration and invasion was gradually increasing, comparing with PBS, M, M_F_, M_FC_, which attributed to Ce6 induced ROS generation and MA induced GSH consumption. As expected, the greatest inhibition of migration and invasion after treatment by BM@M_FC_ and BM@M_FC_C both with 660 nm irradiation, due to additional dBET6 loading causing BRD4 degradation, indicating our nanoparticles could effectively inhibit tumor cells metastasis. Mechanistically, epithelial-mesenchymal transition (EMT) serves as the molecular driver of tumor cells metastasis, BM@M_FC_C might inhibit metastasis by activating EMT process. Therefore, the crucial proteins of EMT (E-cadherin, N-cadherin, and vimentin) after various treatments were investigated by western blotting. As shown in Fig. 4J and Figure S12C-12E, after treated by M_FC_ and M@M_FC_ both with 660 nm irradiation, the expression of E-cadherin (negative correlation with EMT) was gradually increased, while the expression of N-cadherin and vimentin (positive correlation with EMT) were gradually decreased comparing with PBS, M, M_F_, M_FC_ group. Not surprisingly, the BM@M_FC_ and BM@M_FC_C both with 660 nm irradiation group combining Ce6 mediated ROS generation, MA caused GSH depletion, and dBET6 induced BRD4 degradation had the strongest efficiency, indicated that our nanoparticles could effectively inhibit tumor metastasis via EMT process.

### 2.4 *In vitro* immunoregulation of BM@M_FC_C

**Fig. 5:**
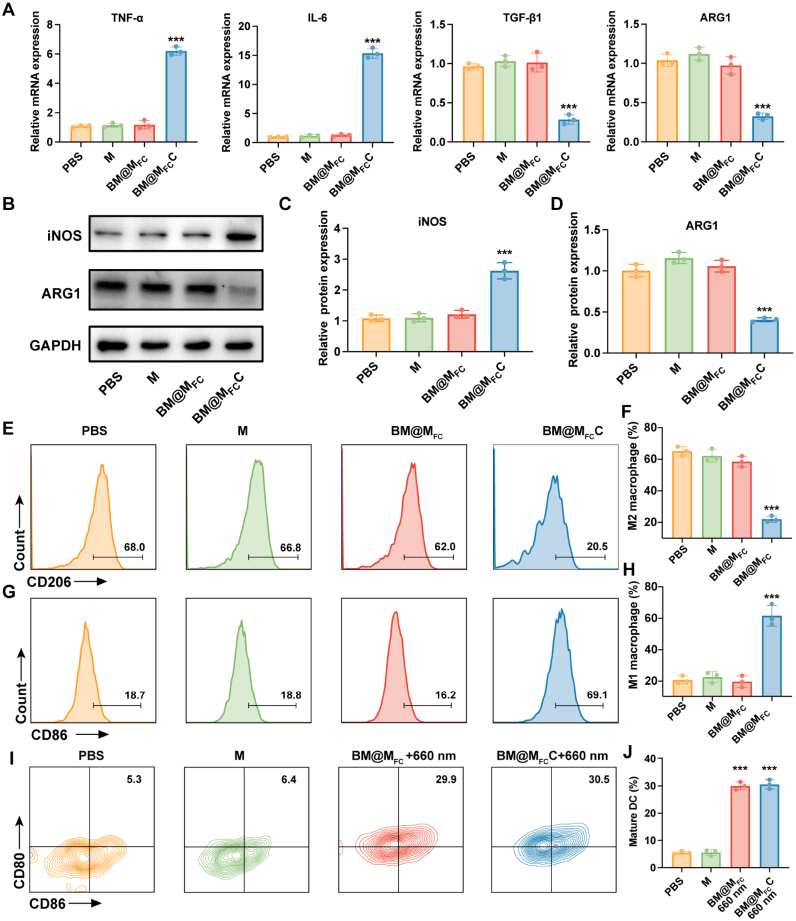
***In vitro* promote macrophage polarization and induce DCs maturation of BM@M_FC_C**. A) mRNA expression of M1 macrophage markers (TNF-α, IL-6) and M2 macrophage markers (TGF-β1, ARG1) in BMDMs cells after treatment by PBS, M, BM@M_FC_ and BM@M_FC_C. B-D) Protein expression of iNOS and ARG-1 in BMDMs cells after different treatments, assessed by western blotting analysis. E-H) Flow cytometry and quantitative analysis of M2 macrophages (CD206^+^) and M1 macrophages (CD86^+^) after different treatments. I, J) Flow cytometry and quantitative analysis of mature DCs (CD80^+^/CD86^+^) after different treatments. Tumor cells were pre-treated with the indicated nanoparticles and exposed to 660 nm irradiation prior to co-culture with DCs. Values are presented as mean ± SEM (n = 3). ∗P < 0.05, ∗∗P < 0.01 and ∗∗∗P < 0.001.

As per the design, BM@M_FC_C would reprogram macrophages from M2 type to M1 type via pH-responsive CaCO_3_ shell dissolution induced acidic TME reverse. Therefore, the IL-4-induced M2 type bone-marrow-derived macrophages (BMDMs) were incubated in a culture medium at pH 6.8 and treated by BM@M_FC_C were investigated by RT-PCR assays. The BMDMs treated with PBS, M and BM@M_FC_ were served as control groups. As shown in Fig. 5A, the M1 markers tumor necrosis factor-α (TNF-α) and interleukin-6 (IL-6) were significantly up-regulated, whereas the M2 markers transforming growth factor beta 1 (TGF-β1) and arginase-1 (ARG-1) were markedly down-regulated in the BM@M_FC_C group compared with other groups, demonstrating that BM@M_FC_C successfully promoted the repolarization of M2 type macrophages to M1 type. Additionally, protein expression of M1 markers inducible nitric oxide synthase (iNOS) and M2 markers (ARG-1) in BMDMs after different treatments were measured by western blotting. As expected, the protein expression of ARG-1 significantly decreased and iNOS obviously increased after treatment by BM@M_FC_C (Fig. 5B–D). More information was obtained by flow cytometry assays to quantitative analysis of macrophage type. The percent of M2 markers (CD206) reduced from 68.0% to less than 20.5% after treatment by BM@M_FC_C, while the M1 markers (CD86) raised from 18.7% to over 69.1% (Fig. 5E–H). Collectively, these results suggested that BM@M_FC_C could reprogram macrophages type from M2 to M1 for enhancing photodynamic immunotherapy.

Considering the combination of efficient PDT activation and BRD4 degradation would efficiently induce apoptosis and ICD to release TAAs for maturing DCs via antigen presentation and further activating photodynamic immunotherapy. The amount of mature DCs (CD80^+^ and CD86^+^ cells) was also investigated by flow cytometry assays. In these experiments, tumor cells were pre-treated with the indicated nanoparticles followed by 660 nm irradiation before co-culture with DCs. As can be seen from Fig. 5I and J, the percentage of mature DCs increased from 5.3% to 29.9% after co-culturing with BM@M_FC_+660 nm-treated tumors cells, compared with PBS and M group, which can be attributed to efficient PDT and dBET6-mediated tumor antigen release. This number was basically unchanged after treatment by BM@M_FC_C+660 nm (30.5%), due to CaCO_3_ shell had litter effect on the maturation of DCs, indicating that BM@M_FC_C could effectively promote DCs maturation for synergistically enhancing photodynamic immunotherapy.

### 2.5 *In vivo* anti-tumor performance of BM@M_FC_C on OSCC

Following the investigation of anti-tumor effect at the cellular level, their *in vivo* therapeutic potential was further evaluated using SCC7 tumor-bearing mice. First, the tissue distribution of BM@M_FC_C after post-intravenous administration was monitored in 4 h, 12 h, 24 h and 48 h via fluorescence imaging, employing DiR-labeled BM@M_FC_C. After the mice were sacrificed, high-resolution ex vivo fluorescence imaging of the major organs (heart, liver, spleen, lung, and kidney) and the tumor was also performed. As illustrated in Fig. S14A and S14B, the fluorescence (DiR-labeled BM@M_FC_C) at tumor site progressively enhanced in 48 h, due to FA receptor-mediated active tumor homing, and passive tumor-selective enrichment via the enhanced permeability and retention effect, demonstrating effective accumulation and retention of BM@M_FC_C in tumor tissue. Although some BM@M_FC_C appeared in the major organs at 4 h, the sustained metabolism caused a negligible content of BM@M_FC_C in these organs after 48 h, which beneficial for further tumor specific therapy (Fig. S14A and S14C). Furthermore, we evaluated the *in vivo* release kinetics of dBET6 and Ce6 from BM@M_FC_C. As shown in Fig. S15, the peak accumulation time of both dBET6 (detected by LC-MS/MS) and Ce6 (detected by fluorescence spectrophotometry) in tumors was 4-8 h post-administration, with an overlapping effective concentration window of 24 h. The superimposed kinetic curves clearly demonstrate the excellent temporal matching of the two drugs, providing a solid foundation for their synergistic anti-tumor effect.

**Fig. 6:**
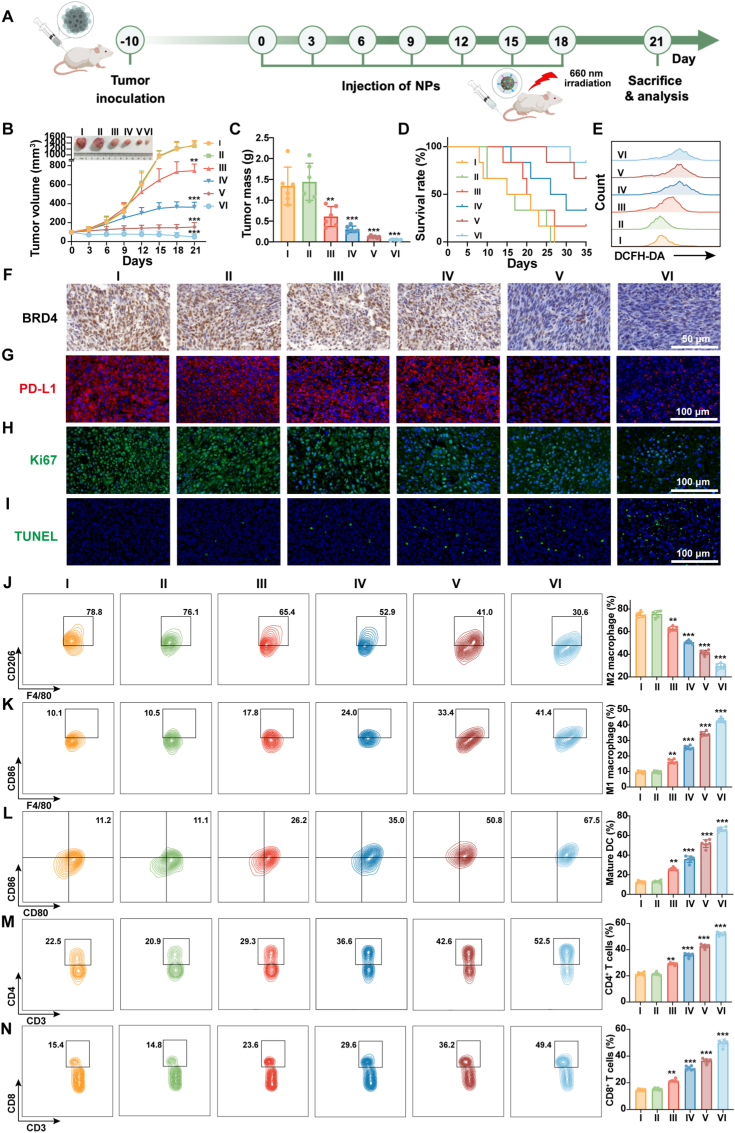
***In vivo* anti-tumor efficacy of BM@M_FC_C in OSCC models.** A) Schematic representation of the treatment protocol in SCC7 tumor-bearing xenograft mice. B) The curves of tumor volume after various treatments against time, and the pictures of tumors obtained at the 21st day. C) The weight of tumors obtained from different groups at the 21st day. D) Survival rate of tumor-bearing mice after different treatments. E) The ROS level in tumor tissues after different treatments at 21 days. F-I) BRD4, PD-L1, Ki67 and TUNEL staining of tumor tissues obtained after 21st day treatment by different formulas. J, K) Flow cytometry analysis the expression of F4/80^+^/CD206^+^ (M2 macrophage markers) and F4/80^+^/CD86^+^ (M1 macrophage markers) in the tumor tissues after different treatments. L) Flow cytometry analysis of the CD86^+^ and CD80^+^ (the markers of mature DCs) in the tumor tissues harvested from the indicated groups. M, N) Flow cytometry analysis of the CD3^+^/CD4^+^ and CD3^+^/CD8^+^ in the tumor tissue of mice after treatment with various formulas. Data are presented as mean ± SEM (n = 5 mice per group, one-way ANOVA followed by Tukey's multiple comparisons test). I: PBS; II: M_FC_; III: M_FC_ + 660 nm; IV: M@M_FC_ + 660 nm; V: BM@M_FC_ + 660 nm; V: BM@M_FC_C + 660 nm. Illumination (660 nm laser, 0.3 W, 10 min) was applied 12 h post intravenous injection. ∗p < 0.05, ∗∗p < 0.01, and ∗∗∗p < 0.001 versus the group I.

In order to evaluate the actual *in vivo* therapeutic efficacy of BM@M_FC_C, the SCC7 tumor-bearing mice were randomly allocated into six experimental groups including: I. PBS (blank control), II. M_FC_ (PDT unactuated), III. M_FC_ with 660 nm irradiation (PDT actuated), IV. M@M_FC_ with 660 nm irradiation (PDT actuated + GSH depletion), V. BM@M_FC_ + 660 nm (PDT actuated + GSH depletion + BRD4 inhibition) and VI. BM@M_FC_C + 660 nm (PDT actuated + GSH depletion + BRD4 inhibition + CaCO_3_ shell coating). The schematic diagram of the treatment protocol for SCC7 tumor-bearing xenograft mice was depicted in Fig. 6A. During the three-week therapeutic period, the average tumor size of different treatment groups was monitored, and the final tumor tissue was separated and weighted. As can be seen from Fig. 6B and C, the tumor growth in M_FC_ with 660 nm irradiation group was inhibited to a certain degree comparing with PBS and M_FC_ group, due to Ce6 induced ROS generation upon 660 nm irradiation for PDT. The treatment efficiency was further enhanced after MA loading (M@M_FC_ with 660 nm irradiation), owe to MA mediated additional GSH consumption. The dual-drug loaded group (BM@M_FC_ with 660 nm irradiation) demonstrated superior therapeutic efficacy compared to mono-therapeutic MA-loaded counterparts, mechanistically attributed to dBET6 additional degraded BRD4 protein. Notably, the BM@M_FC_C with 660 nm irradiation group, which combination of ROS generation, GSH consumption, BRD4 degradation and CaCO_3_ shell induced pH regulation/protection, tumor growth was maximally inhibited as expected. Meanwhile, as the survival curve depicted (Fig. 6D), BM@M_FC_C with 660 nm irradiation treated mice exhibited obviously prolonged survival rate (83.3%, 35 days).

To further delineate the anti-tumor mechanisms of the BM@M_FC_C, flow cytometry analysis was employed to quantitatively assess ROS in tumor tissues after different treatments. As shown in Fig. 6E, significant ROS generation was only observed in groups with both Ce6 and MA (M@M_FC,_ BM@M_FC,_ BM@M_FC_C), indicating that BM@M_FC_C was able to regulate the ROS in tumor tissue via photodynamic process. The GSH levels in tumor tissues were significantly reduced in the M_FC_+660 nm group, which attributed to Ce6-induced ROS generation that consume intrinsic GSH (Fig. S16). Notably, a more pronounced GSH depletion was observed in the MA-containing groups (M@M_FC_+660 nm, BM@M_FC_+660 nm, and BM@M_FC_C+660 nm), due to the combined effect of MA-mediated GSH depletion and ROS-driven consumption. This trend correlates well with the enhanced ROS generation and anti-tumor efficacy observed *in vivo*.

The tumor tissue after different treatments were also obtained and investigated by immunohistochemical staining and immunoblotting, which obviously presented the noticeable inhibition of BRD4 and PD-L1 expression in tumor tissue after treatments by BM@M_FC_C with 660 nm irradiation (Fig. 6F and G). In addition, these tissues were evaluated by Ki-67 staining (typical biomarkers of tumor proliferation) and TUNEL staining (typical biomarkers of tumor apoptosis). Clearly, the BM@M_FC_C with 660 nm irradiation group presented significant down-regulation of Ki-67 and up-regulation of TUNEL (Fig. 6H and I), demonstrating BM@M_FC_C inhibited tumor proliferation and promote apoptosis. Furthermore, we evaluated key immunogenic cell death markers in tumor tissues. As shown in Fig. S17, CRT exposure and HMGB1 release were gradually enhanced in the M_FC_+660 nm, M@M_FC_+660 nm, BM@M_FC_+660 nm, and BM@M_FC_C+660 nm groups compared to the control group. Notably, the BM@M_FC_C+660 nm group exhibited the strongest ICD effect, as evidenced by the highest CRT-positive staining and the lowest HMGB1-positive staining. These results further support that BM@M_FC_C triggers robust ICD to potentiate anti-tumor immunity.

For further evaluated the photodynamic immunotherapy of BM@M_FC_C, the *in vivo* macrophage polarization from M2 type to M1 type was investigated by flow cytometry. As demonstrated in Fig. 6J and K, the percent of M2 marker (CD206) gradually decreasing while the M1 marker (CD86) gradually increasing, in which the BM@M_FC_C with 660 nm irradiation group combined efficient ROS generation, GSH consumption, BRD4 protein degradation, and CaCO_3_ shell induced alkalization/protection had best effect as designed. These results suggested that BM@M_FC_C effectively polarized macrophages from M2 type to M1 type for enhancing photodynamic immunotherapy. Moreover, quantitative analysis of the M1/M2 macrophage ratio confirmed that BM@M_FC_C treatment significantly shifted the balance toward the pro-inflammatory M1 phenotype (Fig. S18A). In addition, the expression of mature DCs markers (CD80 and CD86) was significantly increased after treated by BM@M_FC_C with 660 nm irradiation (Fig. 6L), indicating BM@M_FC_C matured DCs via efficient apoptosis mediated TAAs release for synergistically enhancing photodynamic immunotherapy.

To comprehensively assess the T cells activating potential, caused by M1 macrophages and mature DCs, the helper T cells (CD4^+^ T cells) and effector T cells (CD8^+^ T cells) in allografted mice tumor tissue after different treatments was measured by flow cytometry. As expected, the CD4^+^ and CD8^+^ T cells of BM@M_FC_C + 660 nm treated group had best effect, up to 57.4% and 41.0%, respectively (Fig. 6M and N and Figure S18B). These results indicated that macrophage polarization along with DCs maturation effectively activated T cells for photodynamic immunotherapy. Moreover, we measured the levels of inflammatory cytokines IFN-γ and TNF-α in tumor tissues by ELISA. As shown in Fig. S19, treatment with M_FC_+660 nm, M@M_FC_+660 nm, BM@M_FC_+660 nm, and BM@M_FC_C+660 nm gradually increased IFN-γ and TNF-α production compared to the control group, with the BM@M_FC_C+660 nm group exhibiting the highest levels. These results further support the robust activation of anti-tumor immunity by BM@M_FC_C. Furthermore, the systemic safety assessment revealed maintained body weight profiles (Fig. S20) and absence of histopathological abnormalities in major organs (heart, liver, spleen, lung, and kidney) during and after different treatments (Fig. S21). In addition, no significant difference of alanine transaminase (ALT), aspartate transaminase (AST), urea nitrogen (UREA), creatinine (CREA), or creatine kinase (CK) was observed between PBS and treatment group (Fig. S22), confirming the favorable biosafety of BM@M_FC_C for tumor therapy.

To evaluate whether BM@M_FC_C treatment could establish long-term immune memory and suppress tumor recurrence, we established a SCC7 tumor recurrence model. After the initial treatment regimen (BM@M_FC_C+660 nm or PBS control) for 21 days, the primary subcutaneous tumors were surgically removed, and the mice were monitored for recurrence (defined as tumor volume >200 mm^3^). As shown in Fig. S23A and S23B, all five mice (5/5) in the control group developed significant tumor recurrence within 40 days, with rapidly increasing tumor volumes. In striking contrast, only one out of five mice (1/5) in the BM@M_FC_C+660 nm group showed recurrence, and the recurrent tumor grew much more slowly (Fig. S23C). Consequently, the BM@M_FC_C+660 nm group exhibited a lower recurrence rate and a longer survival period compared to control group (Fig. S23D and S23E). Furthermore, flow cytometry analysis of tumor tissues revealed that BM@M_FC_C treatment markedly increased the infiltration of CD45RA ^+^ CCR7^−^CD4^+^ and CD45RA^+^CCR7^−^CD8^+^ T cells (Fig. S23F–S23H), which are characteristic of effector memory T cells. These results collectively demonstrate that BM@M_FC_C+660 nm treatment not only effectively inhibits primary tumor growth but also establishes long-term anti-tumor immune memory, thereby providing durable protection against tumor recurrence.

### 2.6 *In vivo* single-cell transcriptomics analysis

**Fig. 7:**
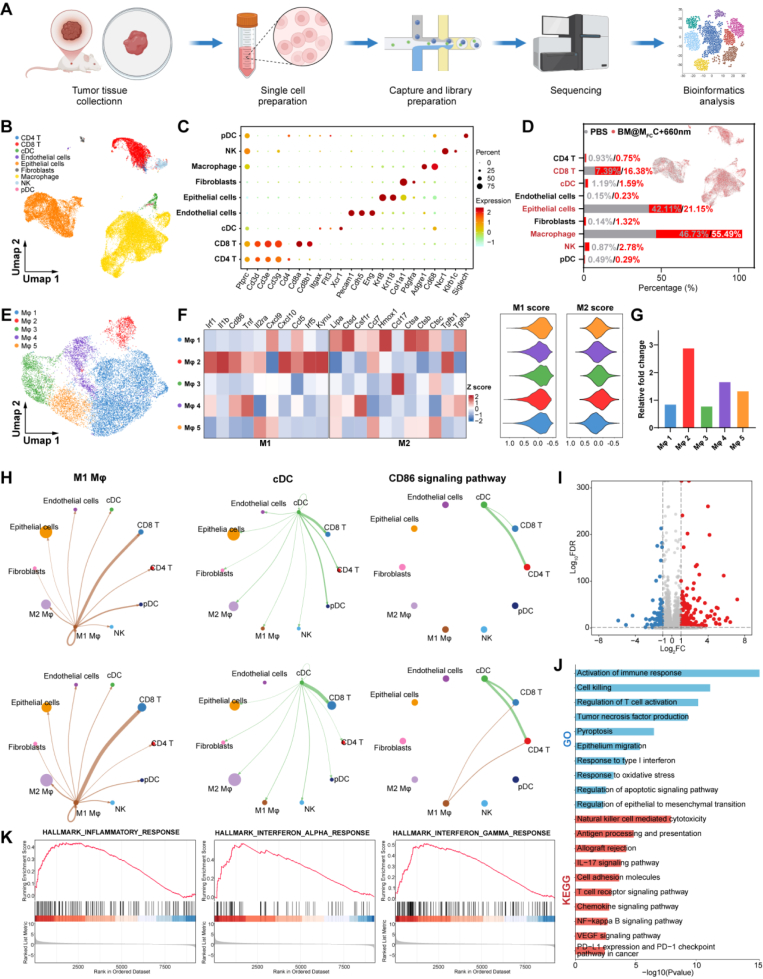
**Single-cell transcriptional profiling of SCC7 allografts following BM@M_FC_C-mediated photodynamic immunotherapy.** A) Schematic illustration of the scRNA-seq of tumor administrated with BM@M_FC_C. B) Uniform manifold approximation and projection (UMAP) plots of 27,031 cells from SCC7 tumor treated with BM@M_FC_C. C) The dot plot shown the highly expressed marker genes across major cell types. D) The stacked histogram depicted the percentage changes of major cell types with or without BM@M_FC_C. E) UMAPs visualization of macrophage subclusters. F) The correlation between each macrophage cluster with M1/M2 signature genes and their M1/M2 scores. G) Histogram chart illustrating the relative changes of each macrophage subcluster before and after treatment. H) Cell-to-cell communication networks inferred with CellChat software. The strength of cell-to-cell interactions was represented with the edge width. I) Volcano plot of differentially expressed genes (DEGs) in tumor epithelial cells (malignant cells) treated with or without BM@M_FC_C. The red, blue, and gray dots represent upregulated genes, downregulated genes, and genes with no significant changes, respectively. J) Gene ontology (GO) and Kyoto Encyclopedia of Genes and Genomes (KEGG) enrichment analysis of DEGs. K) Gene Set Enrichment Analysis (GSEA) results using hallmark genes between tumor epithelial cells treated with or without BM@M_FC_C. (For interpretation of the references to colour in this figure legend, the reader is referred to the Web version of this article.)

To elucidate the mechanisms underlying BM@M_FC_C-mediated photodynamic immunotherapy, we conducted single-cell RNA sequencing (scRNA-seq) on SCC7 tumor tissues after treatments by PBS and BM@M_FC_C with 660 nm irradiation (Fig. 7A). Analysis of 27,031 cells revealed nine distinct populations: epithelial cells (tumor cells), fibroblasts, endothelial cells, macrophages, natural killer (NK) cells, pDCs, cDCs, CD8^+^ T cells, and CD4^+^ T cells, classified using canonical markers (Fig. 7B and C). The BM@M_FC_C + 660 nm treated group exhibited enhanced anti-tumor immunity with increased proportions of CD8^+^ T cells, cDCs, macrophages, and NK cells, accompanied by decreased tumor cell populations compared to untreated controls (Fig. 7D), consistent with previous findings of immunosuppressive microenvironment modulation. Given the critical immunoregulatory role of macrophages in BM@M_FC_C-mediated effects, we identified five macrophage subtypes through reclustering (Fig. 7E). Polarization analysis revealed distinct functional profiles: The Mφ2 subtype displayed predominant M1-like characteristics associated with phagocytic activity and antigen presentation, while Mφ1 exhibited an M2-like type (Fig. 7F). Notably, BM@M_FC_C treatment induced a 2.89-fold increase in M1-polarized Mφ2 macrophages (Fig. 7G), demonstrating successful macrophage repolarization. CellChat analysis of intercellular communication revealed enhanced immune coordination in treated tumors (Fig. 7H). BM@M_FC_C-treated specimens showed strengthened ligand-receptor interactions between M1-like macrophages (Mφ 2) and CD8^+^ T cells, along with intensified cDC-CD8^+^ T cell crosstalk. Particularly robust CD86 signaling emerged from M1-like macrophages and cDCs to both CD8^+^ and CD4^+^ T cell populations, confirming the therapy's capacity for microenvironment reprogramming and immune activation.

Beyond their immunomodulatory functions, our engineered nanoparticles demonstrate direct anti-tumor efficacy through three distinct mechanisms: intracellular ROS generation, GSH depletion, and BRD4 expression suppression. To systematically characterize these therapeutic effects, we performed single-cell transcriptomic profiling on epithelial cell populations. Differential analysis revealed 369 differentially expressed genes (fold changes>2 and false discovery rate<0.05) post BM@M_FC_C treatment, comprising 274 genes upregulated and 95 genes downregulated transcripts (Fig. 7I). Gene ontology (GO) and Kyoto encyclopedia of genes and genomes (KEGG) functional annotation of these differentially expressed genes (DEGs) were primarily enriched in pathways related to tumor-intrinsic modulation, such as cell killing, pyroptosis, response to oxidative stress, regulation of epithelial to mesenchymal transition, PD-L1 expression and PD-1 checkpoint pathway in cancer and immune activation related pathways like activation of immune response, regulation of T cell activation, response to type I interferon, antigen processing and presentation (Fig. 7J). Gene set enrichment analysis (GSEA) further validated the role of BM@M_FC_C treatment in simultaneously inhibiting the malignant type of tumor cells and promoting the activation of anti-tumor immunity (Fig. 7K and S24). Collectively, this single-cell resolution analysis not only corroborates our therapeutic design rationale but also provides unprecedented insights into the coordinated tumor-immune reprogramming induced by BM@M_FC_C.

### 2.7 *In vivo* anti-tumor effect of BM@M_FC_C on primary melanoma and systemic metastases

**Fig. 8:**
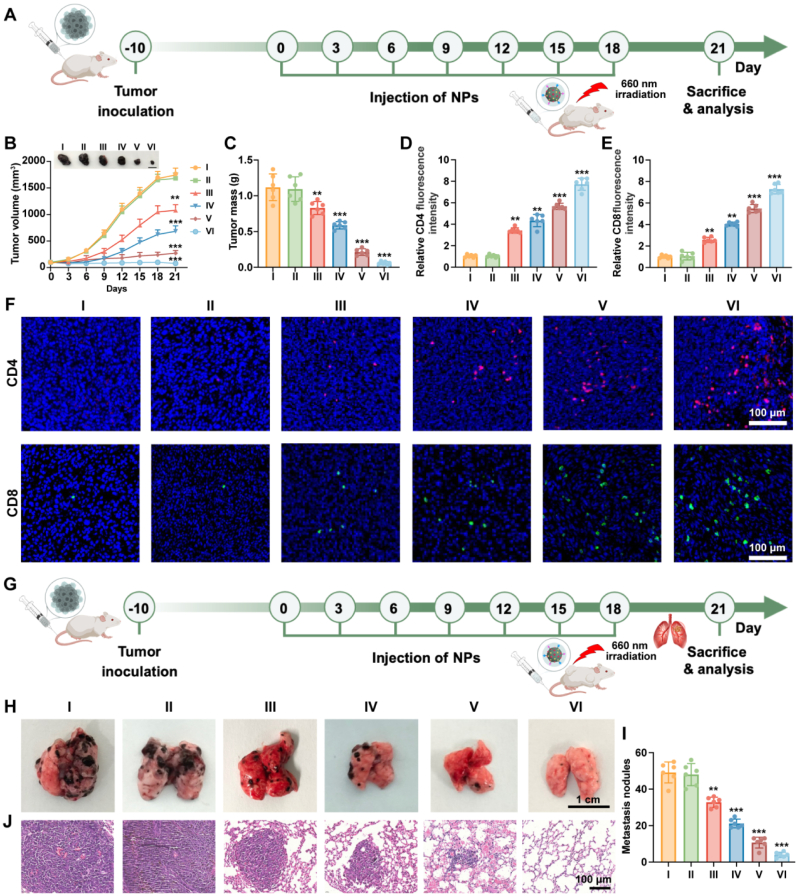
***In vivo* anti-tumor efficacy of BM@M_FC_C in melanoma models.** A) Schematic representation of the treatment protocol in melanoma-bearing xenograft mice. B) The curves of tumor volume after various treatments against time, and the tumors pictures obtained at the 21st day. C) The weight of tumors obtained from different groups at the 21st day. D-F) Fluorescent micrographs and statistics of CD4^+^ and CD8^+^ in the tumor tissues after different treatments. G) Schematic representation of the treatment protocol in melanoma-bearing metastatic mice. H, I) Pictures of the lungs metastases and nodule statistics of those lungs obtained from melanoma model. J) H&E staining of lung tissues from different groups. Data are presented as mean ± SEM (n = 5 mice per group, one-way ANOVA followed by Tukey's multiple comparisons test). I: PBS; II: M_FC_; III: M_FC_ + 660 nm; IV: M@M_FC_ + 660 nm; V: BM@M_FC_ + 660 nm; V: BM@M_FC_C + 660 nm ∗p < 0.05, ∗∗p < 0.01, and ∗∗∗p < 0.001 versus the group I.

After evaluating anti-tumor efficacy of BM@M_FC_C in OSCC models, the tumor therapeutic efficacy was also investigated in melanoma models to comprehensively evaluate the universality of anti-tumor performance. The schematic illustration of the anti-tumor procedure was presented in Fig. 8A. The tumor growth was markedly inhibited after treatment by BM@M_FC_C regardless of the tumor type (Fig. 8B and C**)**. As expected, the CD4^+^ and CD8^+^ T cells in tumor tissues significantly increased in BM@M_FC_C group, demonstrated that our nanoparticles potently activated T cells for photodynamic immunotherapy (Fig. 8D–8F**)**. Furthermore, we assessed the activation status of cytotoxic T lymphocytes by detecting granzyme B^+^ (GZMB^+^) CD8^+^ T cells in tumor tissues. As shown in Fig. S25, treatment with M_FC_+660 nm, M@M_FC_+660 nm, BM@M_FC_+660 nm, and BM@M_FC_C+660 nm gradually enhanced the infiltration of GZMB^+^ CD8^+^ T cells compared to control group, with the BM@M_FC_C+660 nm group exhibiting the most robust increase. These results further confirm the activation of cytotoxic T lymphocyte-mediated anti-tumor immunity by BM@M_FC_C.

Furthermore, *in vivo* ROS generation and BRD4 protein degradation induced anti-metastasis property was further investigated in the melanoma metastasis model. The schematic illustration of the anti-metastasis procedure was shown in Fig. 8G. Massive metastatic nodules were presented in PBS and M_FC_ treated group, while the nodules gradually declined after treatment by other formulas, in which the group binding effective ROS generation and BRD4 degradation (BM@M_FC_C with 660 nm irradiation group) observed the best effect (Fig. 8H and I), demonstrating BM@M_FC_C efficiently inhibited lung metastasis as design. Histopathological evaluation through H&E staining further corroborated these results, with the BM@M_FC_C group maintaining intact pulmonary architecture characterized by preserved alveolar spaces and minimal inflammatory infiltration (Fig. 8J). These results suggested that the treatment of BM@M_FC_C not only inhibited primary tumor growth, but reduced lung metastasis, indicating excellent anti-tumor effect of BM@M_FC_C *in vivo*.

To elucidate the molecular mechanism by which BM@M_FC_C inhibits tumor metastasis, we performed RNA sequencing on lung metastatic tissues from the control group and BM@M_FC_C+660 nm group (Fig. S26A). As shown in Fig. S26B, transcriptomic analysis identified 449 upregulated genes and 411 downregulated genes in the BM@M_FC_C+660 nm group compared to the control. Gene Ontology (GO) enrichment analysis of the upregulated genes revealed significant enrichment in pathways related to activation of immune response, T cell differentiation, regulation of T cell activation, intrinsic apoptotic signaling pathway, and response to hypoxia (Fig. S26C). Kyoto Encyclopedia of Genes and Genomes (KEGG) pathway analysis further indicated enrichment in antigen processing and presentation, the PD-L1/PD-1 checkpoint pathway, apoptosis, cellular senescence, EMT, and TNF signaling (Fig. S26D). Gene Set Enrichment Analysis (GSEA) provided additional mechanistic insights. In the BM@M_FC_C+660 nm group, we observed positive enrichment of pathways involved in transcriptional misregulation in cancer, the PD-L1/PD-1 checkpoint pathway, EMT, and epithelial cell proliferation. Conversely, pathways related to inflammatory response, interferon alpha/beta signaling, T cell activation, and innate immune response activating signaling were negatively enriched in the control group relative to the treatment group (Fig. S26E), indicating that BM@M_FC_C treatment effectively reverses the immunosuppressive state and restores anti-tumor immunity. Collectively, these transcriptomic findings demonstrate that BM@M_FC_C+660 nm inhibits tumor metastasis by inhibiting EMT, enhancing anti-tumor immunity, promoting apoptosis, and modulating key immune-related and oncogenic signaling pathways.

## 3 Conclusion

In summary, we fabricated a specific acidic TME responsive nano-immunoregulator (BM@M_FC_C) for precision photodynamic immunotherapy. The BM@M_FC_C specifically taken up by tumor cells via FA-induced tumor targeting, following with the rapid and efficient generation of ROS to photodynamic apoptosis of tumor cells via Ce6 induced PDT and MA caused GSH depletion. Subsequently, the dBET6 degraded BRD4 synergistically enhanced photodynamic apoptosis, inhibited metastasis and PD-L1 expression. The tumor cells apoptosis further generated many TAAs to mature iDCs and activate T cells for photodynamic immunotherapy. In addition, BM@M_FC_C reshaped the immunosuppressive TME via macrophage polarization and PD-L1 inhibition. Both *in vitro/vivo* results suggested that BM@M_FC_C had remarkable performance to inhibit primary and metastasis tumors. Meanwhile, single-cell RNA sequencing proved BM@M_FC_C induced TME remodeling: increased CD8^+^ T cells, cDCs, and NK cells; M2-to-M1 macrophage polarization; and enhanced intercellular communication. Tumor cell profiling demonstrated BM@M_FC_C downregulated oncogenic pathways and activated immune signatures. Collectively, our BM@M_FC_C nano-immunoregulator provided a promising strategy for precision photodynamic immunotherapy against malignant tumors.

## CRediT authorship contribution statement

**Xiaowei Chang:** Writing – review & editing, Writing – original draft, Validation, Supervision, Formal analysis, Data curation. **Miao Yu:** Writing – original draft, Software, Investigation. **Pan Wei:** Writing – original draft, Resources, Project administration. **Jie Cheng:** Resources. **Yaping Wu:** Writing – review & editing, Validation, Software, Resources.

## Declaration of competing interest

The authors declare that they have no known competing financial interests or personal relationships that could have appeared to influence the work reported in this paper.

## Data Availability

Data will be made available on request.
