# Supplementary material for: Tumor microenvironment responsive nano-immunoregulator for precision cancer photodynamic immunotherapy
Source: Mater Today Bio. 2026 Apr 27;38:103162. doi: 10.1016/j.mtbio.2026.103162 (PMC13142119; doi:10.1016/j.mtbio.2026.103162)
Supplement: Multimedia component 1 [file mmc1.docx]

**Supporting information**

**for**

**Tumor Microenvironment Responsive Nano-Immunoregulator for Precision Cancer Photodynamic Immunotherapy**

Xiaowei Chang^1, #,^ *, Miao Yu^2, #^, Pan Wei^1, #^, Jie Cheng^2^, Yaping Wu^2,^ *

1. Yan’an Medical College, Yan’an University, Yan’an 716000, Shaanxi, China

2. State Key Laboratory Cultivation Base of Research, Prevention and Treatment for Oral Diseases, Nanjing Medical University; Jiangsu Province Engineering Research Center of Stomatological Translational Medicine, Nanjing Medical University; Department of Oral and Maxillofacial Surgery, The Affiliated Stomatological Hospital, Nanjing Medical University, Nanjing 210029, P. R. China

^#^ These authors contributed equally to this work

* Corresponding author: Yaping Wu: wyp_njmu@njmu.edu.cn; Xiaowei Chang: changxw@yau.edu.cn

**1. Materials**

Ammonium hydroxide (25~28 wt% NH_3_•H_2_O), hexadecyl trimethyl ammonium bromide (CTAB), tetraethyl orthosilicate (TEOS), calcium chloride dihydrate (CaCl_2_•2H_2_O) and ammonia bicarbonate (NH_4_•HCO_3_), 1-Ethyl-3-(3-dimethylaminopropyl) carbodiimide hydrochloride (EDC•HCl), N-hydroxysuccinimide (NHS), aminopropyltriethoxysilane (APTES) and were bought from Shanghai Macklin Biochemical Technology Co., Ltd. Methanol, ethyl alcohol and mesitylene were purchased from Sinopharm Chemical Reagent Co., Ltd (Xi’an, China). Maleimide (MA), chlorin e6 (Ce6) and dBET6 was purchased from MedChemExpress LLC. Folic acid (FA) was bought Meryer (Shanghai) Chemical Technology Co., Ltd. All the chemicals were analytical grade and used without further treatment. MilliQ water was prepared using a MilliQ system (Bedford, MA, America).

5× SDS loading buffer (#P0015), BCA Protein Assay Kit (#P0011), DAPI (#C1002), and Cell Complete Lysis Buffer for Western and IP (#P0037) were bought from Beyotime Biotech (China). High-sig ECL Western Blotting Substrate (#180−501) was supplied by Tanon Biotech (China). Recombinant murine M-CSF (#315-02) and recombinant murine IL-4 (#214-14) were bought from PeproTech Inc. (USA). DMEM (#11965092), RPMI-1640 medium (#1187509), and FBS (#10270−106) were bought from ThermoFisher (USA). The anti-GAPDH (#2118), anti-iNOS antibody (#13120), anti-Arginase-1 antibody (#93668), anti-CD206 antibody (#91992), anti-N-Cadherin antibody (#13116), and anti-E-Cadherin antibody (#3195) were obtained from Cell Signaling Technology (USA). The antibody against BRD4 (#67374-2-Ig), Vimentin (#60330-1-Ig), PD-L1 (CD274; #66248-1-Ig), FOLR1 (#23355-1-AP) and c-Myc (#67447-1-Ig) were purchased from Proteintech (Wuhan, China). The Ki-67 (#ab15580) antibody was obtained from Abcam (UK). The BB700-conjugated F4/80 antibody (#746070), PE-Cy7-conjugated CD86 antibody (#560582), BV421-conjugated CD80 antibody (#562611), FITC-conjugated CD3 antibody (#561827), BV605-conjugated CD4 antibody (#563151), PE-Cy5.5-conjugated CD8 (#561109), and FVS780-conjugated Live/ Dead fixable viability staining kit (#565388) were purchased from BD Bioscience (USA). APC-conjugated CD206 antibody (#141708) was obtained from Biolegend (USA).

**2. Preparation process of BM@M_FC_C nanoparticles**

2.1 Preparation of amino modified mesoporous silica nanoparticles (MSN-NH_2,_ M_N_)

As to prepare the BM@M_FC_C nanoparticles, the mesoporous silica nanoparticles (MSNs) were first prepared by the classical CTAB-templated, base-catalyzed sol-gel method based on the previous work of our laboratory. The pH value of 200 ml deionized water was adjusted to approximately 11 with 10.6 ml ammonium hydroxide (25~28 wt% NH_3_•H_2_O). The temperature was raised to 50 ℃, and then 224 mg CTAB was added. After the CTAB was completely dissolved, 1.2 ml TEOS were added dropwise with rapid stirring. After 2 h, the mixture was sat at room temperature for 12 h, then centrifuged and washed thoroughly with distilled water and ethanol. As-synthesized MSNs were dispersed in ethanol by sonication for 30 min, followed by addition of 10 ml mixed solvent (1:1, water and mesitylene). The mixture was placed in the autoclave, and kept at 140 °C for 4 days without stirring. The resulting white powder was washed with ethanol and water five times each. Then, the surfactant templates (CTAB) were removed by extraction using acidic methanol (1.8 mL of HCl/80 ml of methanol, 36 h) at 65 °C, which were further centrifuged, washed several times with ethanol and dried under vacuum for 24 h to obtain MSNs.

In order to modified amino group on the surface of MSNs, the as-synthesized 200 mg MSNs were dispersed in 20 ml toluene, and then 200 μl APTES was added into the suspension and stirred for 8 h at 110 ℃ under N2 atmosphere. Then the mixture was centrifuged, washed with ethanol, and vacuum dried to obtain amino modified MSNs (M_N_).

2.2 Preparation of FA and Ce6 co-modified MSNs (M_Fc_)

The FA and Ce6 co-modified MSNs were synthesized through amidation reaction. In detail, FA (2 mg) and Ce6 (2 mg) were co-dispersed in 10 ml mixed solvent (1:1, water and DMSO), then EDC•HCl (27 mg) and NHS (7 mg) was added into the suspension and stirred for 1 h. After that, the as-synthesized 20 mg M_N_ nanoparticles were added to this mixture and stirred for 12 h. Then, the mixture was centrifuged, washed with H_2_O and ethanol, and vacuum dried to obtain FA and Ce6 co-modified MSNs (MSN-FA/Ce6, M_FC_).

2.3 Preparation of dBET6 and MA co-loaded nanoparticles (BM@M_FC_)

The dBET6 (2 mg) and MA (2 mg) was dissolved in 1 ml DMSO, then added to a solution of M_FC_ (10 mg in 10 ml H_2_O), the mixture was stirred at room temperature for 12 h. After that, the mixture was centrifuged, washed with H_2_O and freeze drying to obtain BM@M_FC_ nanoparticles.

2.4 Preparation of final product (BM@M_FC_C)

First, 20 mg CaCl_2_•H_2_O, 10 mg BM@M_FC_ were dissolved in 20 ml ethanol in a glass bottle covered by an aluminum foil which was punctured with several pores. Then, the bottle was put into a vacuum drying chamber containing 200 mg dry ammonia bicarbonate (NH_4_•HCO_3_). After 12 h, the BM@M_FC_C nanoparticles were obtained and could be separated by centrifugation and vacuum drying.

## 3. Characterization

Transmission electron microscopy (TEM) images were recorded on a Lorenz Transmission Electron Microscope. For the TEM observation, samples were obtained by dropping 10 μl of solution onto carbon-coated copper grids. All the TEM images were visualized without staining. The zeta potentials were measured by a Delsa™ Nano C particle analyzer (Beckman Coulter, USA) running Delsa Nano software and using 4 mW He-Ne laser operating at a wavelength of 633 nm and avalanche photodiode (APD) detector. The infrared (IR) spectra were measured by Nicolet iS50 FT-IR. Elemental analysis was obtained using a Hitachi S-4800 scanning electron microscope equipped with energy dispersive spectrometer (EDS). The ultraviolet-visible (UV-Vis) spectra were measured with dilute aqueous solution in a 2 mm thick quartz cell using a Hitachi U-2910 spectrophotometer. The N_2_ adsorption-desorption isotherms were obtained on a Microtrac BEL BELSORP-Max at 77 K under continuous adsorption condition.

**4. Cell Culture**

HOK, SCC7, and B16-F10 cells were obtained from the American Type Culture Collection (ATCC, USA). These cells were cultured in RPMI-1640 or DMEM medium, supplemented with 10% fetal bovine serum (FBS), at 37°C in a humidified incubator with 5% CO₂.

Primary bone marrow-derived macrophages (BMDMs) were extracted from C57BL/6 mice and cultured in DMEM supplemented with 4.5 g/L glucose, 2 × 10^−3^ M L-glutamine, 10% FBS, and 1% penicillin/streptomycin at 37°C in a 5% CO_2_-humidified environment. Briefly, the femurs and tibias of six-week-old C57BL/6 mice were dissected; subsequently, the bone marrow cavities were flushed with 1 × PBS and treated with red blood cell lysis buffer. Following centrifugation of the cell supernatant, the cells were resuspended in DMEM containing M-CSF at a concentration of 40 ng/mL. After a cultivation period of seven days, the BMDMs were collected and utilized for subsequent experimental procedures.

Additionally, primary bone marrow-derived dendritic cells (BMDCs) were isolated from the bone marrow monocytes of C57BL/6 mice and maintained in complete RPMI-1640 medium (10% FBS and 1% penicillin/streptomycin) under identical conditions of 5% CO2 at 37°C. In a similar manner, the bone marrow cells were harvested, resuspended, and cultured in complete RPMI-1640 medium supplemented with M-CSF (20 ng/mL) and IL-4 (10 ng/mL). On Day 6, the immature dendritic cells (iDCs) were collected for further experimental investigations.

**5. Cellular uptake**

HOK cells and SCC7 cells were seeded into a 24 well-plate and cultured overnight. Then, cells were incubated with PBS, rhodamine B (RhB) labeled BM@M_C_C, or BM@M_FC_C for indicated conditions. For fluorescence microscope detection, the cells were washed with PBS buffer, fixed with 4% paraformaldehyde, stained with DAPI, and photographed under a fluorescence microscope. For flow cytometry analysis, the cells were harvested and analyzed using a BD LSRFortessa (BD, USA) flow cytometer.

**6. Western blotting**

After treated with different formulas, cells were collected and lysed using Cell Complete Lysis Buffer supplemented with protease inhibitors for 15 min on ice. Subsequently, the cell lysates underwent centrifugation at 4°C for a duration of 10 minutes to remove the pellets. The protein concentrations were quantified utilizing the BCA protein assay reagent kit. Following the determination of the concentration, a 5× loading buffer containing bromophenol blue was incorporated into the protein-containing supernatant. For the purpose of immunoblotting analysis, the cellular proteins were separated using 10% SDS-PAGE and subsequently transferred onto PVDF membranes. The protein-blotted membranes were incubated with primary antibodies overnight at 4°C. After this incubation period, secondary antibodies were applied and incubated at 37°C for 1 hour. Finally, the detection of the bands was accomplished using the ECL kit.

**7. CCK8 assay**

Cells were seeded into 96-well plates under the concentration of 3000 cells per well. After adherence, the cells were treated with indicated nanoparticles or regents. After indicated time points, 10 µL of Cell Counting Kit 8 (CCK8) solution was added into each well of the plates and incubated at 37°C for another 1 h. Then the absorbance at 450 nm of the plates was monitored by a multiplate reader (BioTek, USA).

**8. Cell migration and invasion assay**

Cell migration or invasion ability was detected using wound healing assay or transwell assay, respectively. In the wound healing assay, the cells after indicated treatment were cultured in a 6-well plate until they reached an optimal confluence of approximately 80%. Subsequently, a sterile 200 µL pipette was employed to create a scratch across the monolayer of cells. Following washing with PBS, the migration of the cells was monitored at multiple time intervals using an inverted fluorescence microscope (Carl Zeiss, Germany). In the transwell assay, 24-well transwell chambers (Corning) were pre-treated with Matrigel (#354234, Corning, USA) and allowed to incubate overnight at 37°C. The cancer cells subjected to treatment were then placed in the upper chamber containing serum-free medium, while the lower chamber was filled with complete medium supplemented with fetal bovine serum (FBS). After a duration of 24 hours, cells remaining in the upper chambers were carefully removed with a cotton swab. The transwell chambers were subsequently fixed using methanol for 10 minutes and stained with crystal violet for an additional 20 min. After thorough rinsing with PBS, images of the chambers were captured using an inverted fluorescence microscope (Carl Zeiss, Germany). The number of migrating or invading cells was quantified and analyzed using Image J software for comparison.

**9. RNA extraction and real-time PCR analysis**

Total RNA extraction was isolated via RNAiso Plus (#9108, Takara, Japan) reagent. Following this, reverse transcription of RNA and real-time PCR were conducted employing the PrimeScript RT reagent kit and RT-PCR kit (Takara, Japan). The relative expression levels of mRNA were determined through the comparative Ct method, with GAPDH serving as the internal control. The primers targeting the genes of interest were designed and synthesized by Sangon Biotech (China), detailed gene-specific primers were listed as follows: TNF-α Forward: GGTATGAGCCCATCTATC, Reverse: GCAATGATCCCAAAGTAG; IL6 Forward: CTGCAAGAGACTTCCATCCAG, Reverse: AGTGGTATAGACAGGTCTGTTGG; TGFB1 Forward: CTGGCCTGCTGCTGCTGCTGCTG, Reverse: GGCATGTGGCTTCTATGGTGG; ARG1 Forward: CTCCAAGCCAAAGTCCTTAGAG, Reverse: GGAGCTGTCATTAGGGACATCA.

**10. ATP release assay**

To evaluate ATP release as an additional immunogenic cell death (ICD) marker, SCC7 cells were seeded in 6-well plates at a density of 5 × 10⁵ cells per well and allowed to adhere overnight. The cells were then treated with different formulations (M_FC_, M@M_FC_, BM@M_FC_, and BM@MFCC) with or without 660 nm irradiation (10 mW/cm², 5 min) according to the experimental design. After 24 h of incubation, the cell culture supernatants were collected and centrifuged at 1,000 × g for 5 min to remove cell debris. ATP levels in the supernatants were measured using a commercial ATP bioluminescence assay kit (Beyotime, China) following the manufacturer’s instructions. Briefly, 100 μL of supernatant was mixed with an equal volume of ATP detection reagent, and luminescence was immediately recorded using a microplate reader (Bio-Rad, USA). ATP concentrations were calculated from a standard curve and normalized to total protein content.

**11. Hematoxylin-Eosin (HE), immunohistochemical (IHC) staining, and immunofluorescence (IF) Staining**

HE, IHC, and IF staining procedures were conducted on tumor tissues and major organs that had been preserved in formalin and subsequently embedded in paraffin. The resultant samples were sectioned to a thickness of 4 µm in preparation for the subsequent staining processes. For HE staining, slides were deparaffinized and stained with hematoxylin and eosin. For IHC staining, slides were deparaffinized, and heat-induced epitope retrieval (HIER) was performed using EDTA for 15 min at 95°C. Sections were incubated with primary and corresponding secondary antibodies, followed by visualization with a DAB detection kit and counterstaining with hematoxylin. For IF staining, the slides were deparaffinized and permeabilized. Sections were blocked, incubated with primary antibodies overnight, and then with fluorescently labeled secondary antibodies. Nuclei were counterstained with DAPI. Stained sections were visualized and photographed using a fluorescence microscope (Carl Zeiss, Germany).

**12. Flow cytometry assay**

The cells or samples after indicated treatment were collected and prepared for single-cell suspension. The resulting cell suspension underwent live/dead viability staining, after which Fc receptor blocking was conducted. Upon completion of the membrane surface antigen staining, the cells were washed and then resuspended in a staining buffer. For the anti-CD206 staining, cells underwent fixation and permeabilization subsequent to the membrane surface antigen staining. The analysis of single or multicolor stained cells was carried out using a BD LSRFortessa flow cytometer (BD, USA). The raw data files generated were further analyzed and visualized using FlowJo v10.8 software (BD, USA).

**13. Animals and tumor models**

This study was approved by the Institutional Animal Care and Use Committee of Nanjing Medical University (Approval No. IACUC-2407203 on 2024-07-13). All procedures performed were in accordance with the ethical standards of the institutional research committee. All mice were housed in a specific pathogen-free (SPF) facility with a 12-hour light-dark cycle and had unrestricted access to food and water. Female C3H/He mice and C57BL/6 nude mice (5-6 weeks of age) were provided by Beijing Vital River Biological Technology Co., Ltd. (China). Additionally, four-week-old male C57BL/6 mice were provided by and Nanjing Medical University Animal Center.

In order to generate SCC7-derived allograft, a total of 5 × 10^5^ cells were inoculated subcutaneously into the right flank of C3H/He mice. Tumor volume was assessed daily, calculated using the formula (Length × width × width)/2. Once the mean tumor volume reached 100 mm^3^, the mice were randomly grouped into six groups (six mice per group) and administrated with indicated treatment. The entire therapeutic regimen spanned a duration of 21 days. The body weights and tumor sizes of the mice were monitored and documented every three days. Additionally, the cardiac, hepatic, pulmonary, renal, and splenic tissues were harvested for HE, IHC, or IF staining and further analysis. For survival evaluation, the mice were similarly divided into six groups (n = 6) as previously mentioned. After administering the same therapeutic regimen, the survival rate of SCC7-tumor-bearing mice was assessed using the Kaplan-Meier method and analyzed with the Log-rank test on day 35.

To evaluate long‑term immune memory and tumor recurrence after BM@M_FC_C treatment, a SCC7 tumor recurrence model was established. SCC7 cells (5 × 10⁵) were subcutaneously inoculated into the right flank of C3H/He mice. When the mean tumor volume reached approximately 100 mm³, the mice were randomly divided into two groups (n = 5 per group) and treated with either PBS (control) or BM@M_FC_C+660 nm irradiation according to the same therapeutic regimen described above. After 21 days of treatment, the primary subcutaneous tumors were surgically excised under sterile conditions. Tumor recurrence was monitored post-surgery, and significant recurrence was defined as a tumor volume exceeding 200 mm^3^. Tumor volumes were measured every three days, and mouse survival was recorded. For analysis of long‑term effector memory T cells, tumor tissues were collected at the endpoint, and flow cytometry was performed to detect CD45RA⁺CCR7⁻CD4⁺ and CD45RA⁺CCR7⁻CD8⁺ T cells. Data were analyzed using the Kaplan‑Meier method with the Log-rank test.

B16-F10-tumor-bearing mice were established to assess the therapeutic efficiency of these nanoparticles on primary tumor growth and metastasis. As described earlier, C57BL/6 mice were subcutaneously injected with 10^6^ cells and divided into six groups when the tumor volume reached approximately 100 mm^3^. The mice were then treated with the same therapeutic regimen used for the SCC7-tumor-bearing mice. After 21 days of administration, the mice were euthanized, and the tumors were collected for further analysis. Additionally, another set of C57BL/6 mice were intravenously injected with 5 × 10^5^ cells to develop a lung metastasis model. Following 21 days of treatment administration, these mice were also euthanized, and their lungs were collected and imaged to evaluate tumor metastasis.

**14. ELISA analysis of cytokines in tumor tissues**

To evaluate the activation of antitumor immunity, the levels of inflammatory cytokines IFN-γ and TNF-α in tumor tissues were measured by enzyme-linked immunosorbent assay (ELISA). After the final treatment, SCC7 tumor tissues were collected from each group (n = 3 per group), thoroughly washed with ice-cold PBS, and homogenized in RIPA lysis buffer containing protease inhibitors. The homogenates were centrifuged at 12,000 × g for 15 min at 4 °C, and the supernatants were collected for cytokine quantification. The concentrations of IFN-γ and TNF-α were determined using commercial ELISA kits (e.g., R&D Systems, USA) according to the manufacturer’s instructions. Absorbance was read at 450 nm using a microplate reader (Bio-Rad, USA), and cytokine levels were calculated based on standard curves and normalized to total protein concentration.

**15. Bulk RNA sequence and analysis**

Lung metastatic tissues were collected from B16-F10 tumor-bearing mice in the control group (PBS) and the BM@M_FC_C+660 nm treatment group (n = 3 per group). Total RNA was extracted using TRIzol reagent (Invitrogen, USA) according to the manufacturer’s protocol. RNA concentration and purity were assessed using a NanoDrop 2000 spectrophotometer (Thermo Fisher Scientific, USA), and RNA integrity was verified by 1% agarose gel electrophoresis. Sequencing libraries were prepared using the NEBNext® Ultra™ RNA Library Prep Kit for Illumina (NEB, USA) following the manufacturer’s instructions. Briefly, mRNA was enriched from total RNA using oligo(dT) magnetic beads, fragmented, and reverse transcribed into cDNA. The cDNA library was subjected to end repair, A‑tailing, adapter ligation, and PCR amplification. The resulting libraries were sequenced on an Illumina NovaSeq 6000 platform (Illumina, USA) with paired-end 150 bp reads.

Raw sequencing reads were filtered to remove adaptor sequences and low‑quality reads using fastp (v0.23.0). Clean reads were aligned to the mouse reference genome (mm10) using HISAT2 (v2.2.1). Gene expression levels were quantified by featureCounts (v2.0.1), and differentially expressed genes (DEGs) were identified using DESeq2 (v1.32.0) with the criteria of |log_2_(fold change)| > 1.5 and adjusted P value (Padj) < 0.05. Gene Ontology (GO) enrichment analysis and Kyoto Encyclopedia of Genes and Genomes (KEGG) pathway analysis of upregulated DEGs were performed using the clusterProfiler R package (v4.0.5). Gene Set Enrichment Analysis (GSEA) was conducted using the fgsea R package with the hallmark gene sets (h.all.v7.4.symbols.gmt) from the Molecular Signatures Database (MSigDB). Enriched pathways were considered significant when Padj < 0.05 and false discovery rate (FDR) < 0.05.

**16. Single-cell RNA sequencing (scRNA-seq)**

For scRNA-seq, the SCC7 tumor tissues of three different mice with or without BM@MFCC + 660 nm irradiation were collected. The scRNA-seq library was prepared using the DNBelab C4 series Single Cell RNA Library Preparation Kit (MGI Tech Co., Ltd.) by Shanghai Biotechnology Corporation. Briefly, the fresh tumor tissues were enzymatically digested into single cells. Then, the single-cell suspensions were converted to barcoded scRNA-seq libraries through steps including droplet encapsulation, emulsion breakage, mRNA captured beads collection, reverse transcription, cDNA amplification, and purification. cDNA production was sheared to short fragments with 300-500 bp, and indexed sequencing libraries were constructed according to the manufacturer’s protocol. Qualification was performed using Qubit ssDNA Assay Kit (Thermo Fisher Scientific) and Agilent Bioanalyzer 2100. All libraries were further sequenced by the DNBSEQ-T7 sequencing platform with pair-end sequencing. The sequencing reads contained 30-bp read 1 (including the 10-bp cell barcode 1, 10-bp cell barcode 2 and 10-bp unique molecular identifiers (UMI)), 100-bp read 2 for gene sequences and 10-bp barcodes read for sample index.

The sequencing data were processed using an open-source pipeline (https://github.com/MGI-tech-bioinformatics/DNBelab_C_Series_scRNA-analysis-software). Briefly, all samples were performed sample de-multiplexing, barcode processing, and single-cell 3’ unique molecular identifier (UMI) counting with default parameters. Processed reads were then aligned to GRCh38 genome reference using STAR (2.5.1b). Valid cells were automatically identified based on the UMI number distribution of each cell by using the “barcodeRanks()” function of the DropletUtils tool to remove background beads and the beads that had UMI counts less than the threshold value. Finally, we used PISA to calculate the gene expression of cells and create a gene x cell matrix for each library.

**17. Statistical analysis**

All quantitative data presented in this study was shown as mean ± standard deviation (SD) of at least three independent experiments. Statistical analysis was compared with Student’s t-test (two-tailed), one-way analysis of variance (ANOVA). The overall survival (OS) rate of tumor patients was estimated using Kaplan–Meier method and compared with Log-rank test. The difference between groups was considered statistically significant for *P < 0.05, very significant for **P < 0.01, and most significant for ***P < 0.001. All statistical analyses were performed using Graph-Pad Prism 7.0 (GraphPad Software, USA) or SPSS 22.0 (IBM, USA).


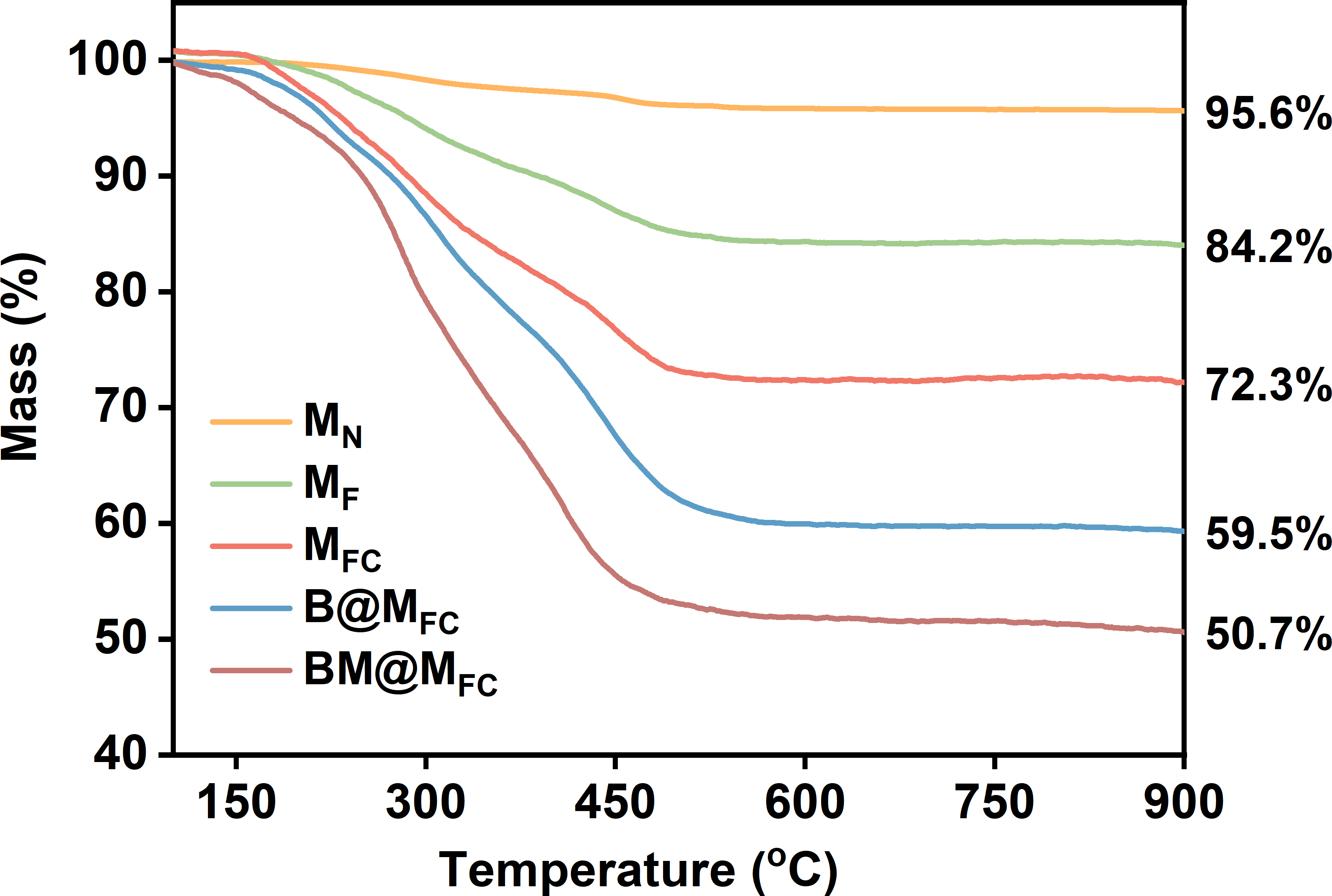


**Figure S1.** The thermogravimetric analysis results of M, M_F_, M_FC_, B@M_FC_ and BM@M_FC_.


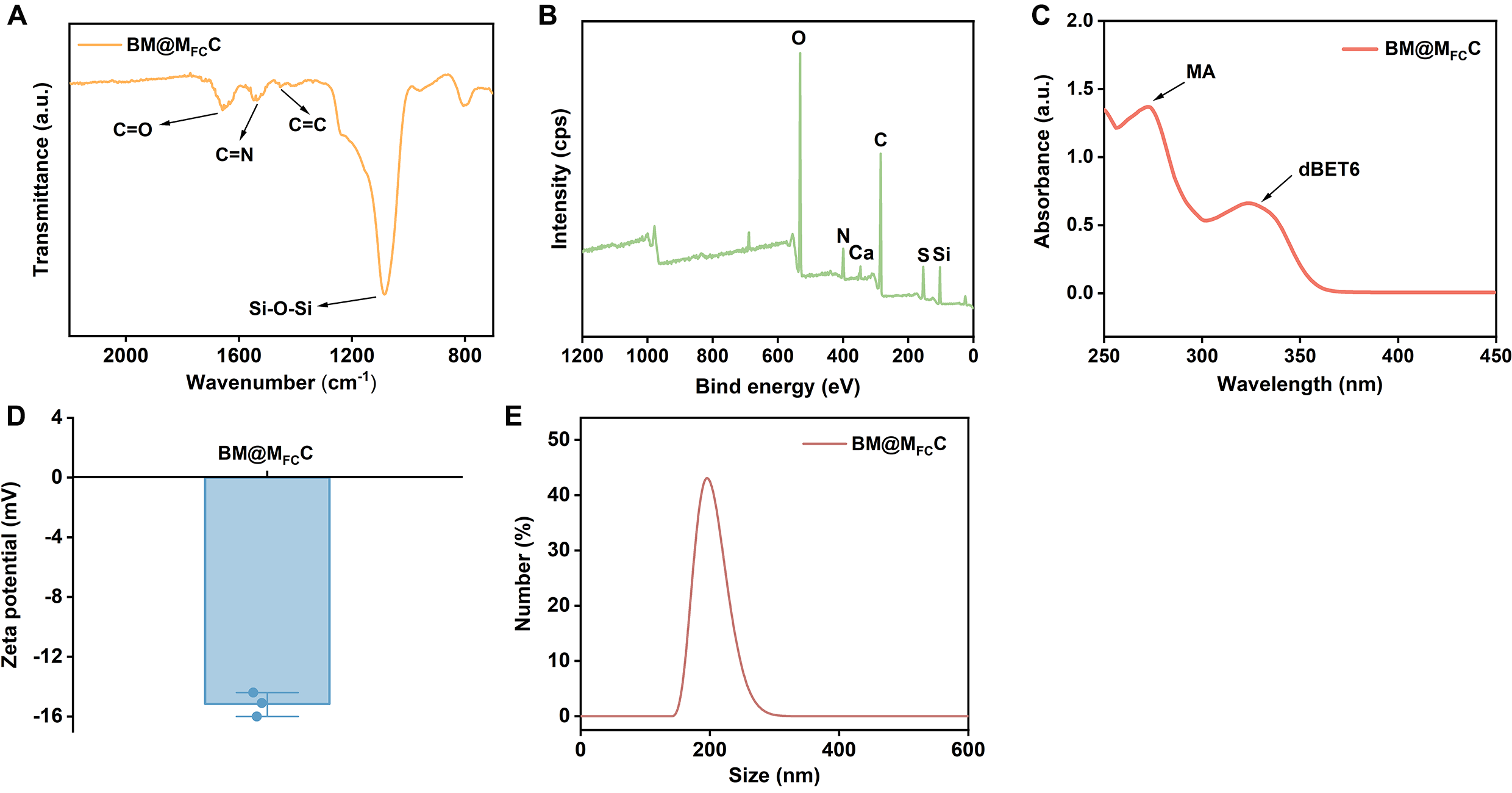


**Figure S2.** Characterization of BM@M_FC_C. A) Fourier transform infrared (FTIR) spectra. B) Full-spectrum X-ray photoelectron spectroscopy (XPS). C) UV-vis spectrometry. D) Zeta potential. E) DLS.


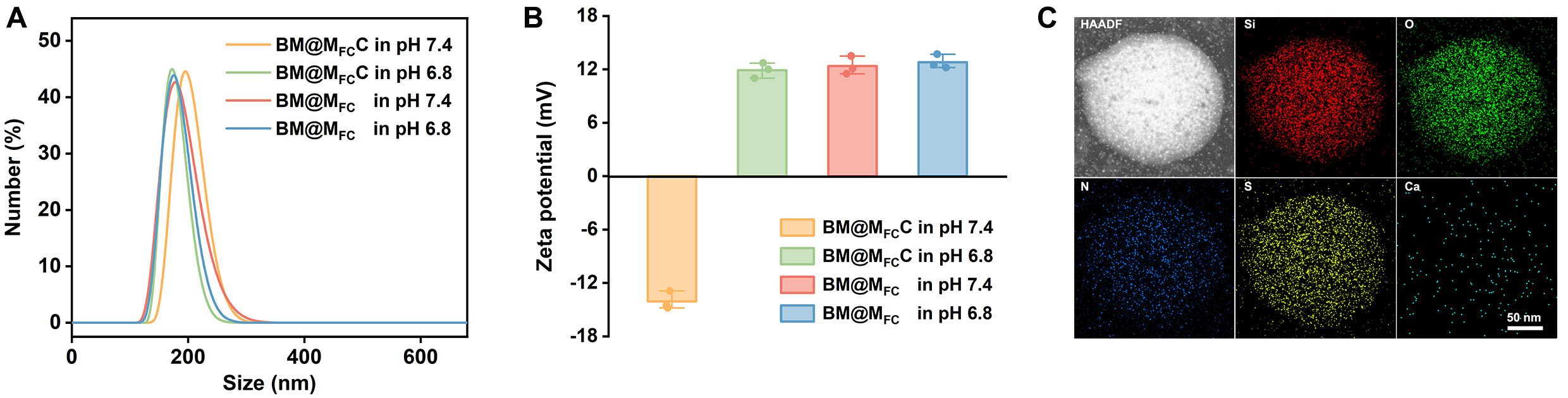


**Figure S3.** A) Hydrodynamic diameter and (B) Zeta potential of BM@M_FC_ and BM@M_FC_C at pH 6.8 and pH 7.4. C) Elemental mapping analysis of BM@M_FC_C after incubation in pH 6.8.


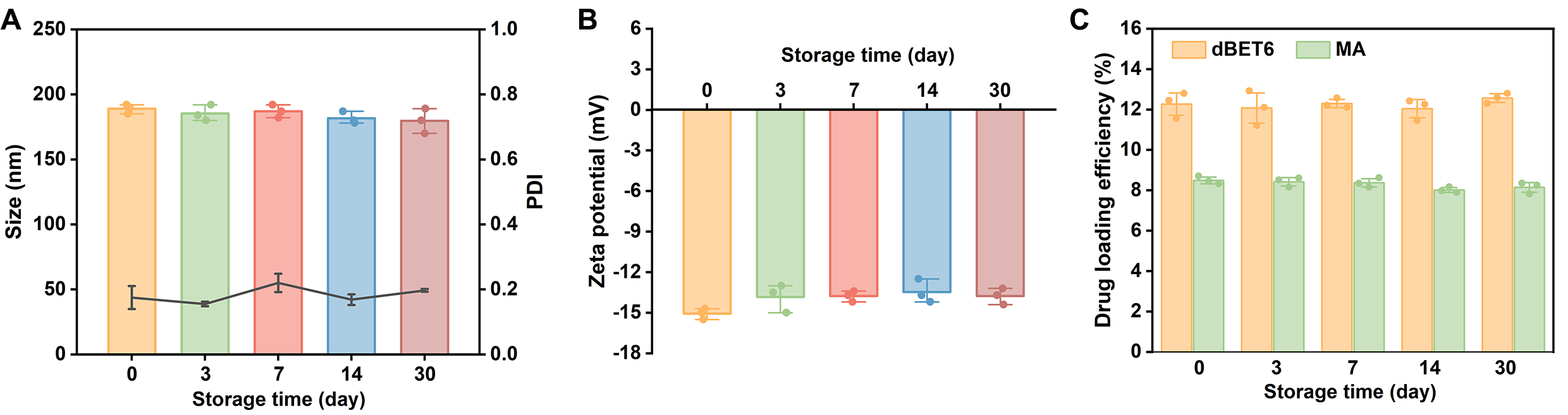


**Figure S4.** Storage stability of BM@M_FC_C nanoparticles at 4 °C for 30 days. A) Hydrodynamic diameter and PDI. B) Zeta potential. C) Relative loading efficiency of dBET6 and MA.


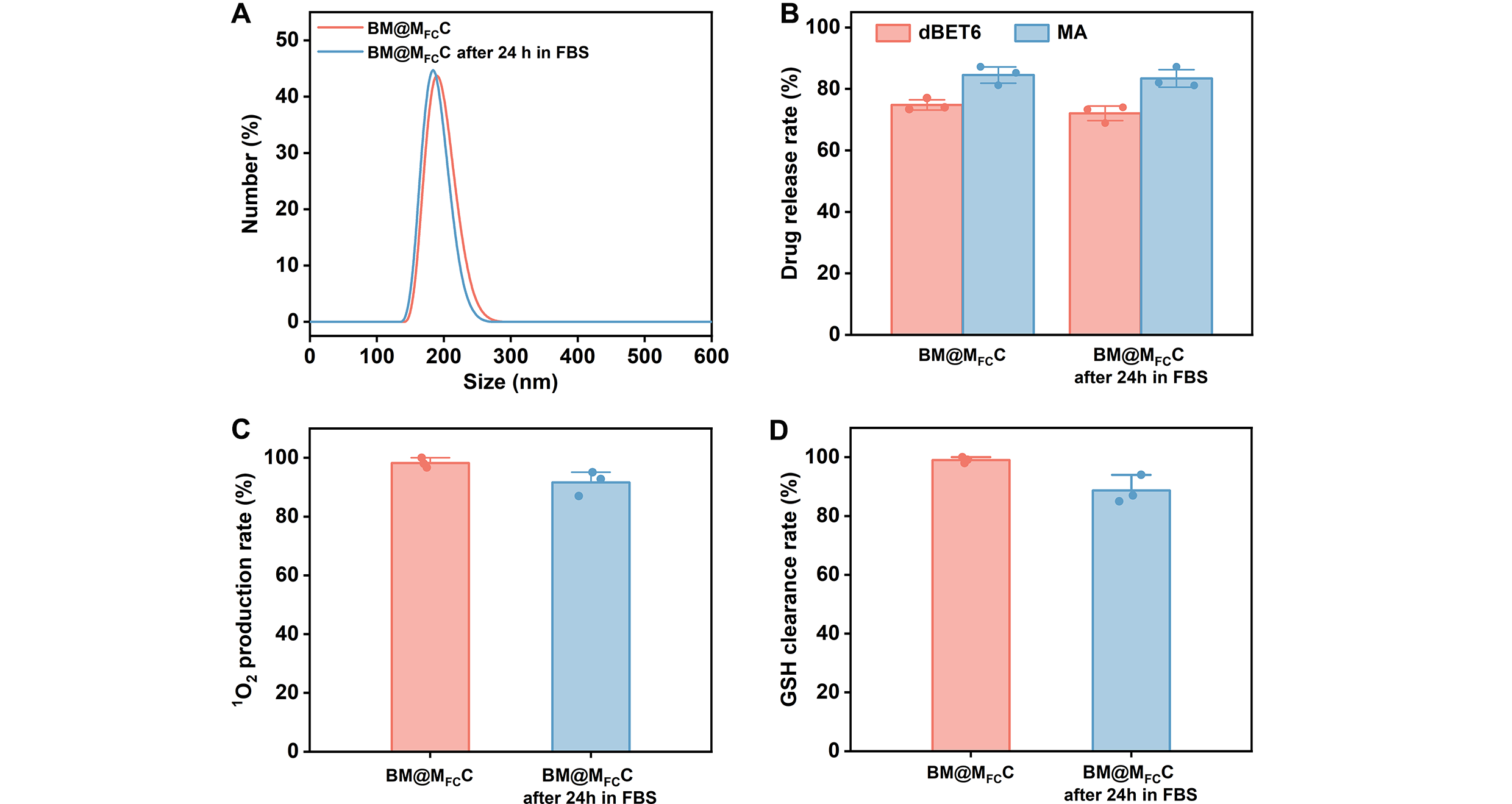


**Figure S5.** Serum stability of BM@M_FC_C nanoparticles in 10% FBS at 37 °C for 24h. A) The average hydrodynamic size, B) Cumulative drug release percentage, C) ^1^O_2_ generation rate and D) GSH clearance rate of BM@M_FC_C before and after 24h incubation in FBS.


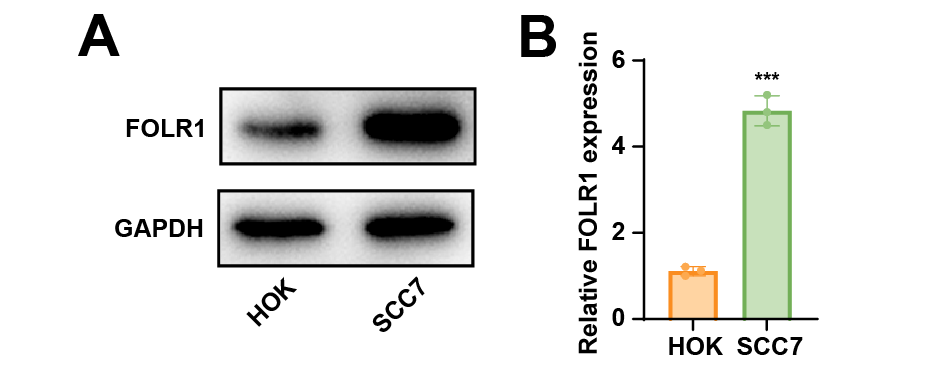


**Figure S6.** A) Representative Western blot analysis of FOLR1 protein expression in human oral keratinocytes (HOK) and SCC7 oral squamous cell carcinoma cells. GAPDH was used as a loading control. B) Quantitative analysis of relative FOLR1 protein levels normalized to GAPDH.


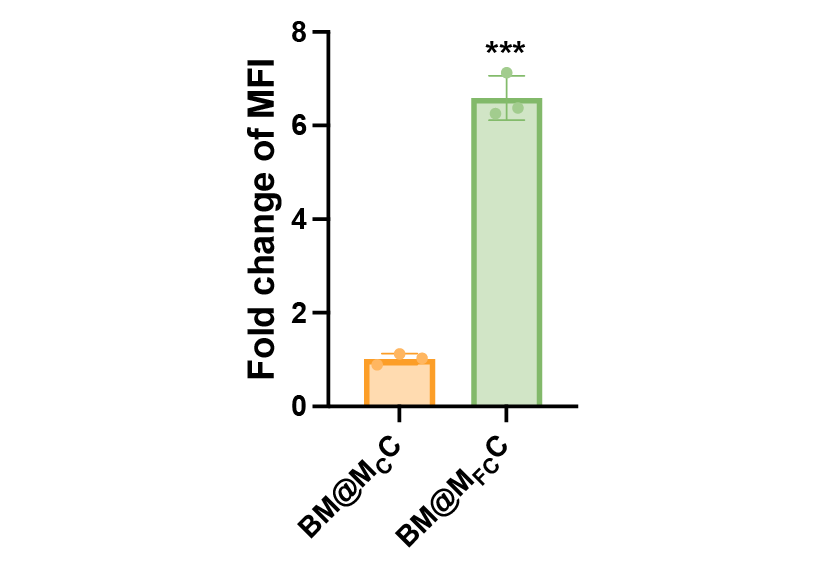


**Figure S7.** Fold change in median fluorescent intensity of HOK cells and SCC7 cells treatment with RhB-labeled BM@M_C_C and BM@M_FC_C, respectively.


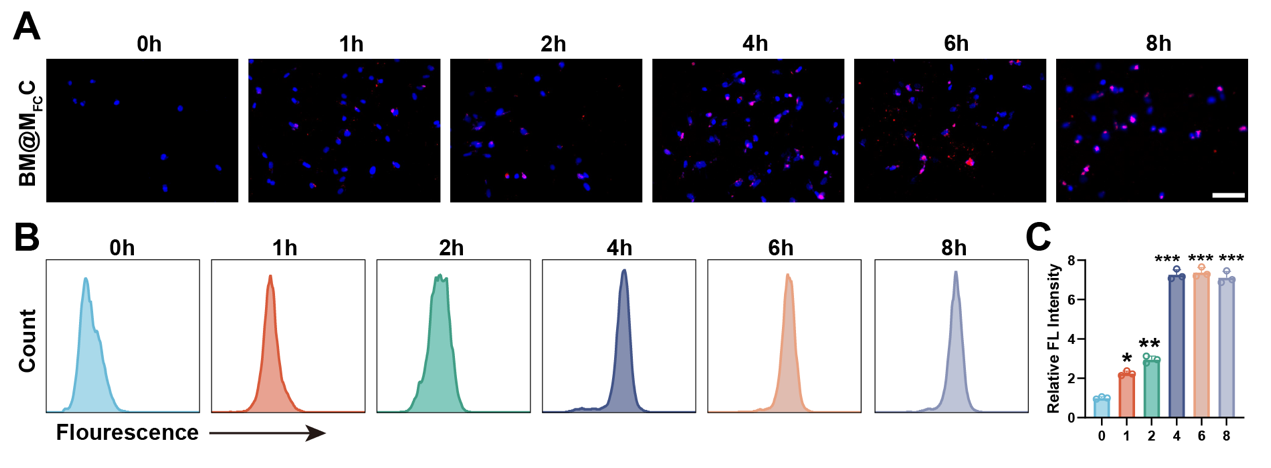


**Figure S8.** A) Confocal fluorescence microscopy images of SCC7 cells after incubation with rhodamine B (RhB)-labeled BM@M_FC_C for the indicated time points; nuclei were pre-labeled with DAPI. Scale bar: 100 μm. B) Flow cytometry analysis of SCC7 cells incubated with RhB-labeled BM@M_FC_C for the indicated time points. C) Fold change in median fluorescence intensity of SCC7 cells following incubation with RhB-labeled BM@M_FC_C.


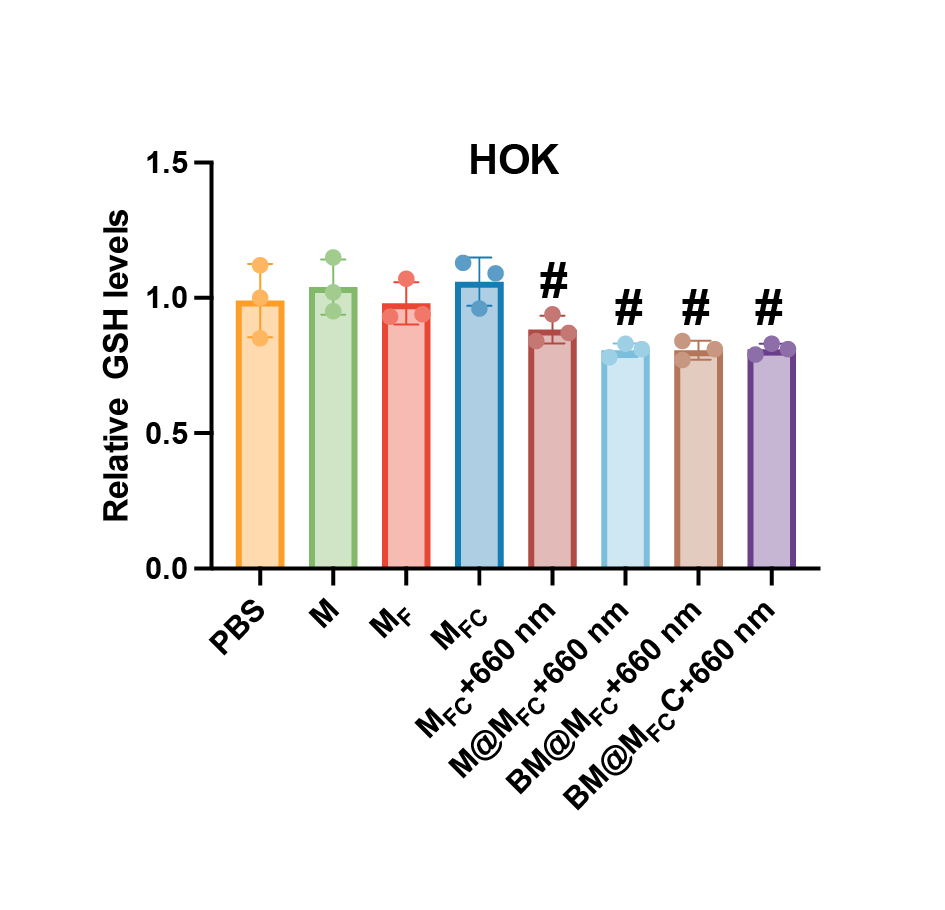


**Figure S9.** Relative GSH levels in HOK cells after different treatments. Cells were treated with PBS, M, M_F_, M_FC_, M_FC_+660 nm, M@M_FC_+660 nm, BM@M_FC_+660 nm, or BM@M_FC_C+660 nm. GSH levels were measured and normalized to the PBS control group. Data are presented as mean ± SD (n = 3).


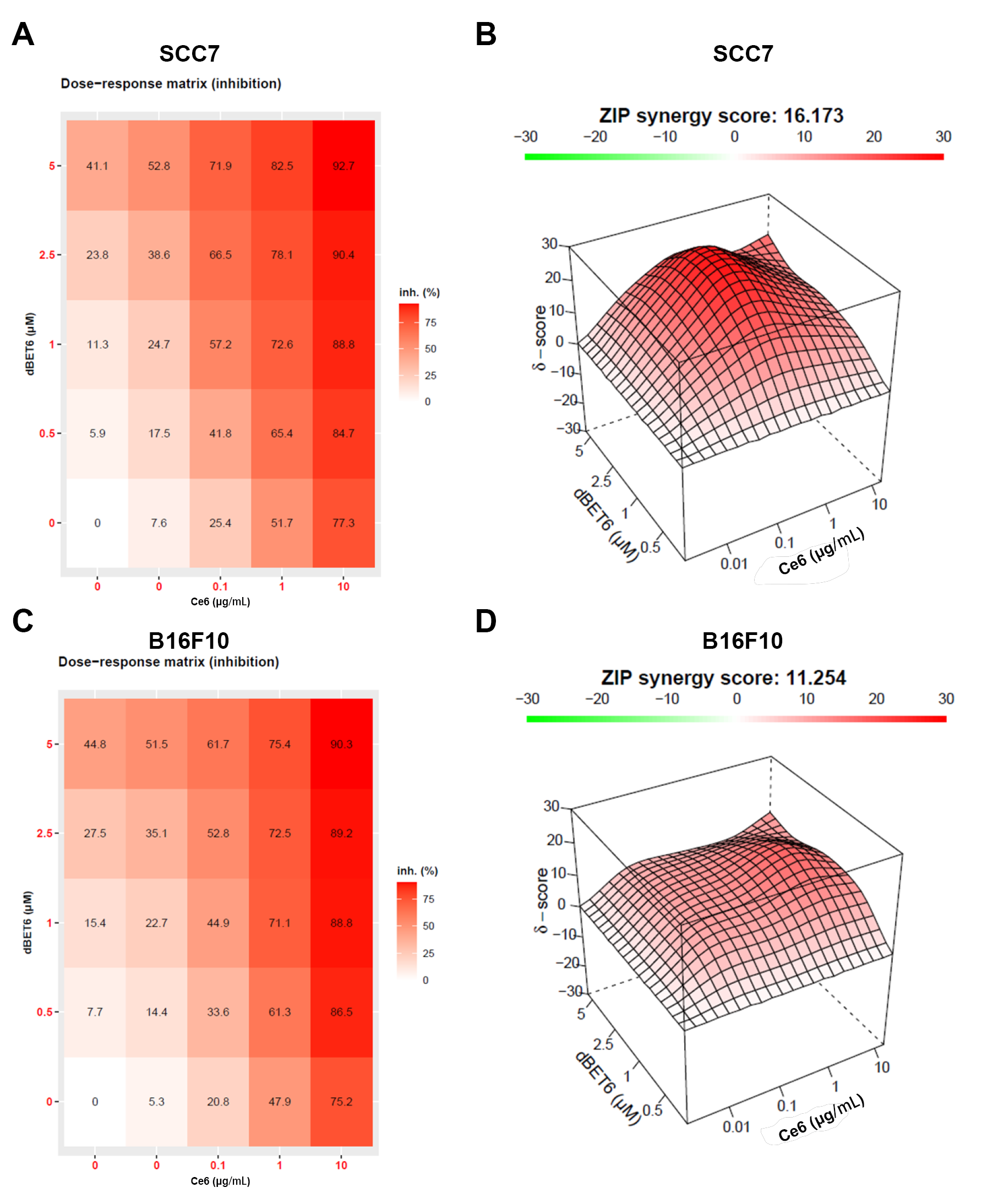


**Figure 10.** A, C) Dose-response matrices showing the inhibition percentage of cell viability in (A) SCC7 and (C) B16F10 cells treated with various concentrations of dBET6 and Ce6 alone or in combination for 48 hours. (B, D) Three-dimensional synergy landscapes calculated using the ZIP (Zero Interaction Potency) synergy model. The ZIP synergy scores were 16.173 for SCC7 cells (B) and 11.254 for B16F10 cells (D), indicating strong synergistic interactions between dBET6 and Ce6 (scores > 10 typically indicate strong synergy).


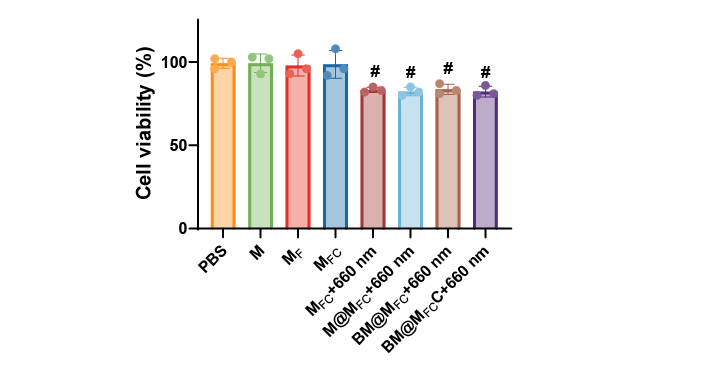


**Figure S11.** Cell viability of HOK cells after incubated with PBS, M, M_F_, M_FC_, M_FC_ + 660 nm irradiation, M@M_FC_ + 660 nm irradiation, BM@M_FC_ + 660 nm irradiation and BM@M_FCC_ + 660 nm irradiation.


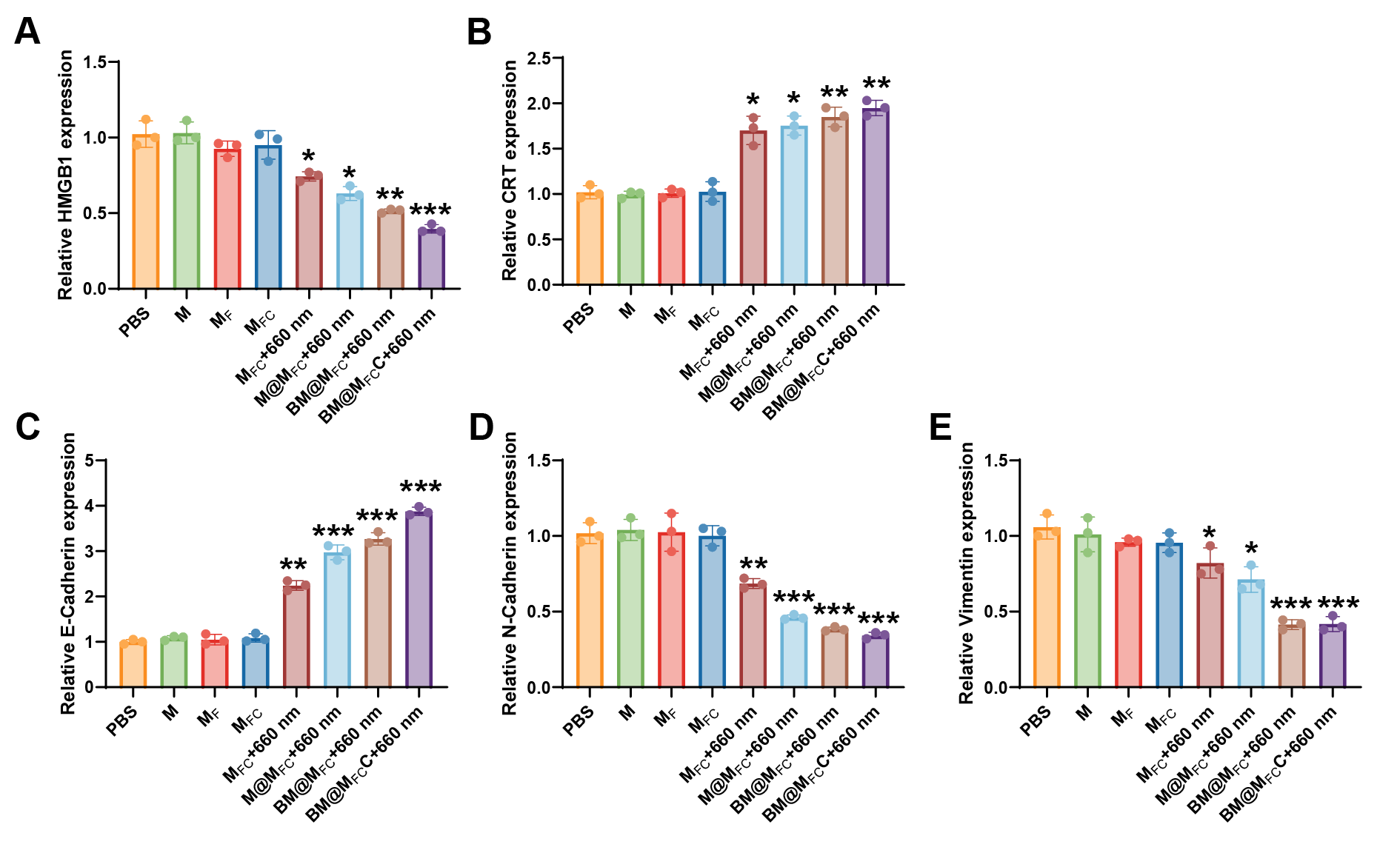


**Figure S12.** Western blot quantification of protein expression in cells treated with various formulations. Relative expression levels of (A) HMGB1, (B) CRT, (C) E-cadherin, (D) N-cadherin, and (E) Vimentin were normalized to loading control.


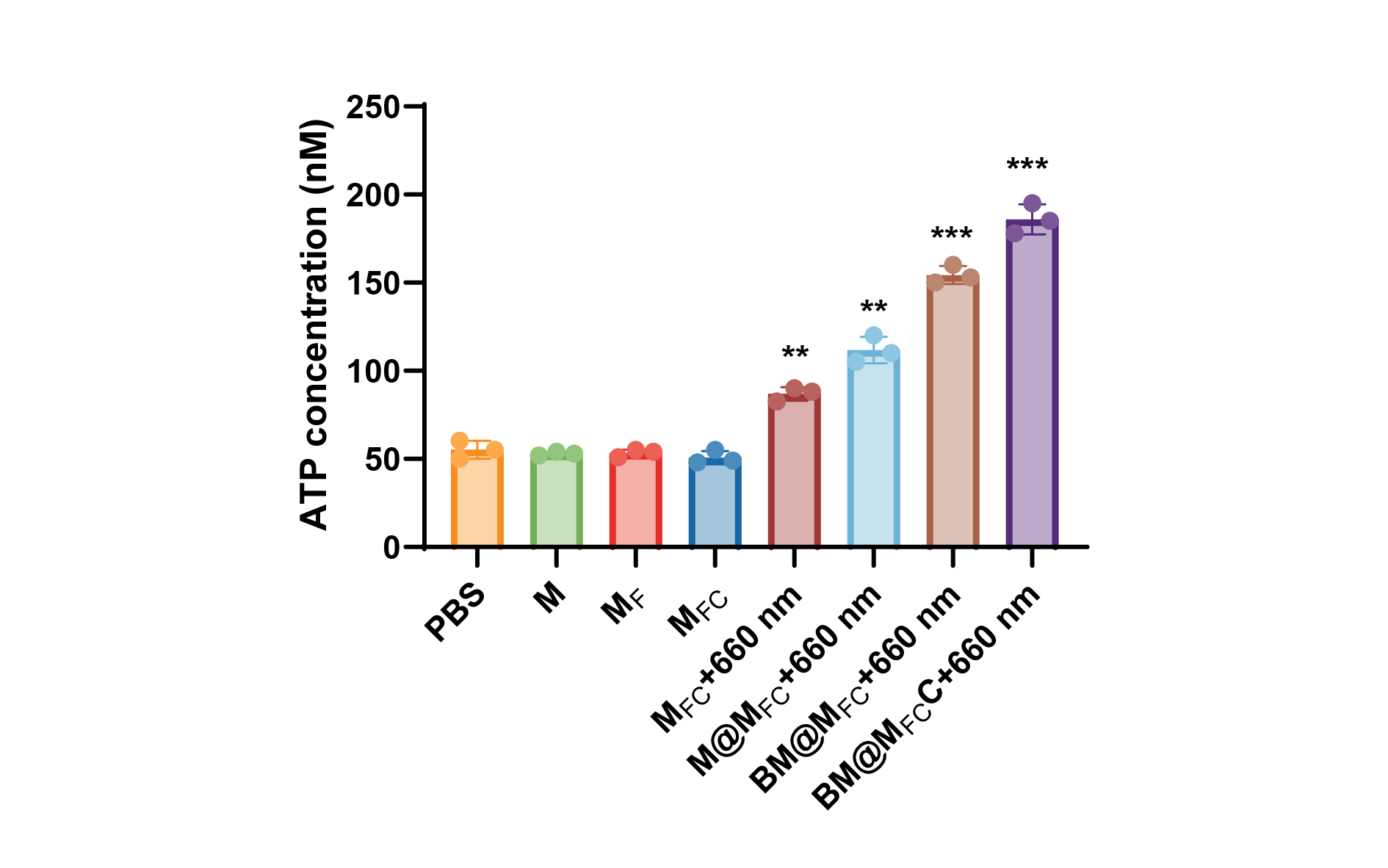


**Figure S13.** Extracellular ATP concentration measured in culture supernatants after treatment with various formulations.


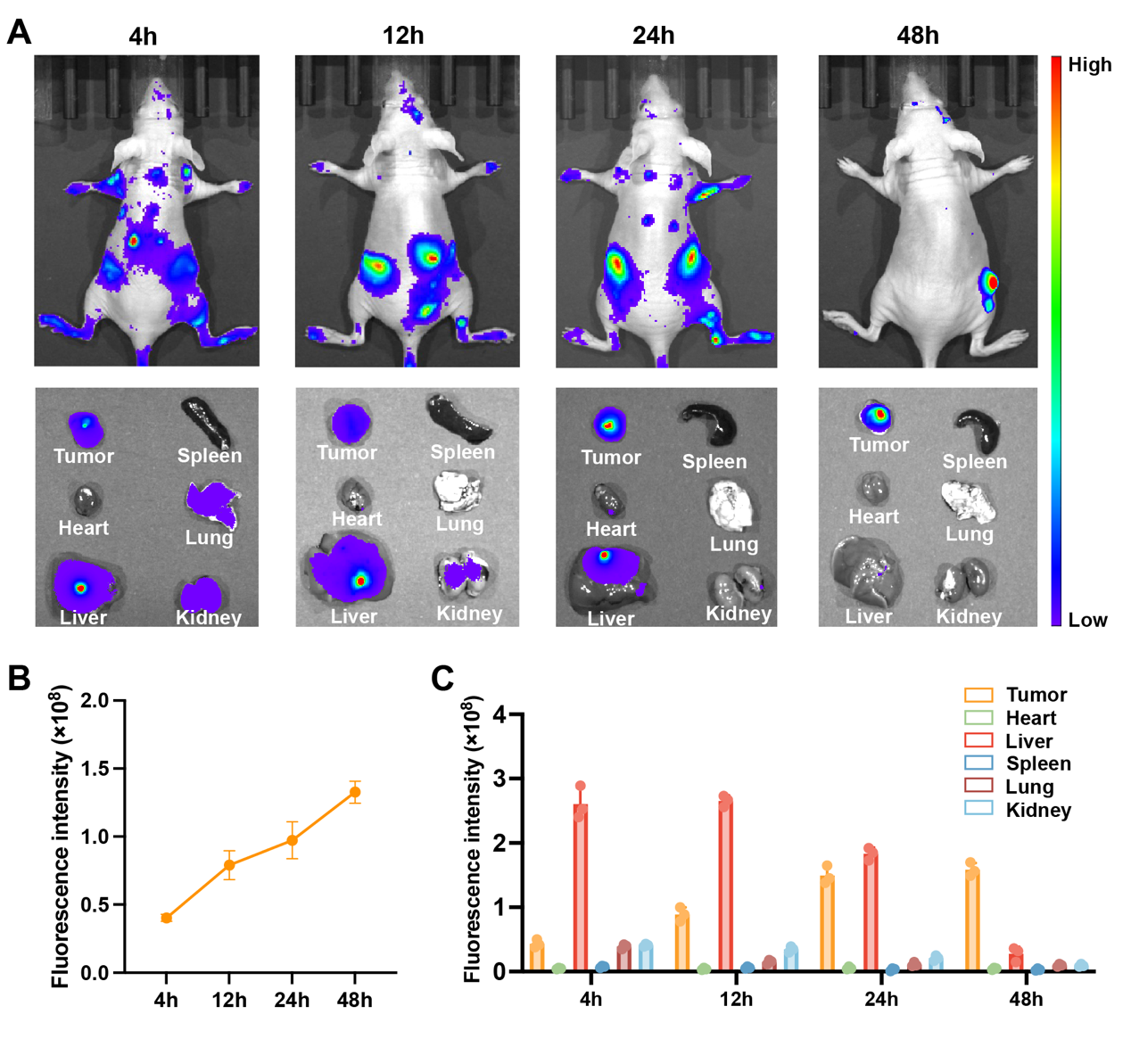


**Figure S14.** A) *In vivo* fluorescence images of the tumor-bearing mice at 4 h, 12 h, 24 h, and 48 h after injection of DiR-labeled BM@M_FC_C as well as *ex vivo* fluorescence images of tumor and major organs. B) Quantitative analysis of tumor fluorescence intensity over time. C) Fluorescence intensity quantification in major organs (tumor, heart, liver, spleen, lung, and kidney) at 4 h, 12 h, 24 h, and 48 h post-injection.


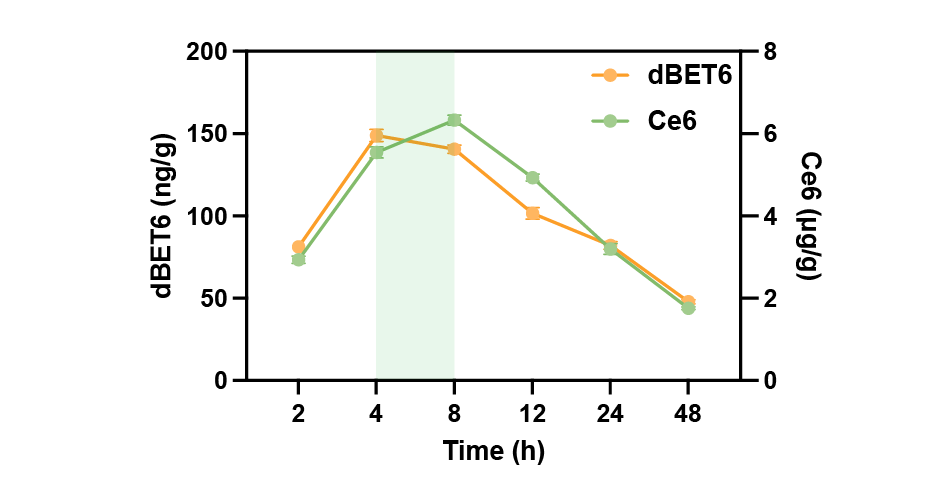


**Figure S15.** *In vivo* tumor accumulation and release kinetics of dBET6 and Ce6. dBET6 concentrations were quantified by LC-MS/MS (left Y-axis, orange line), while Ce6 concentrations were measured by fluorescence spectrophotometry (right Y-axis, green line).


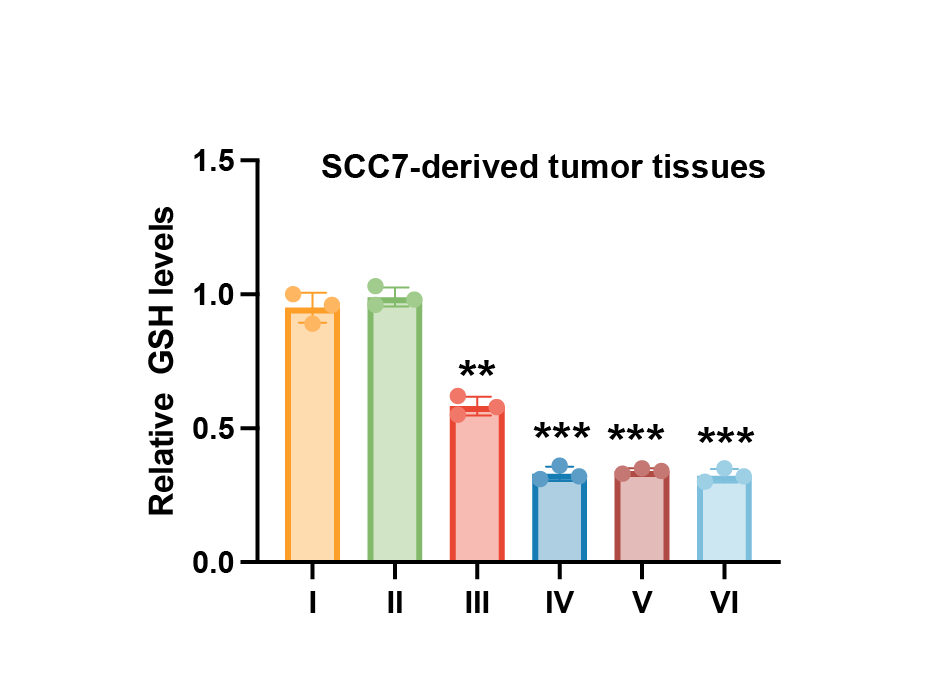


**Figure S16.** Relative GSH levels in SCC7 tumor tissues after different treatments. Data are presented as mean ± SD (n = 3).


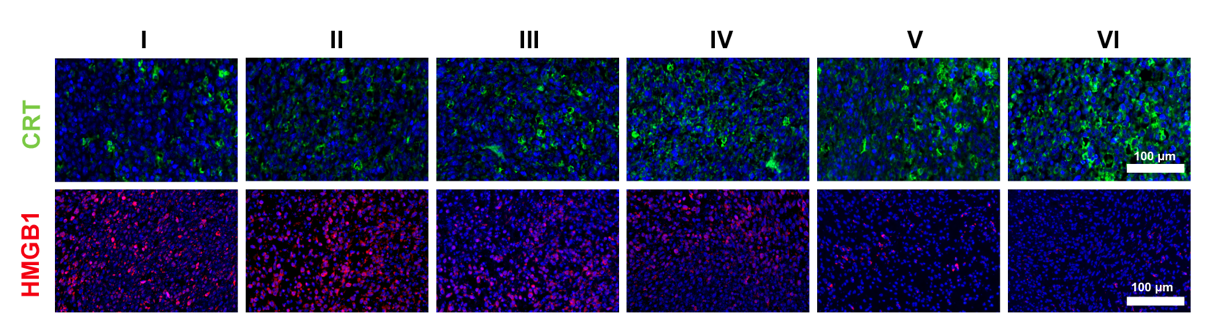


**Figure S17.** Immunofluorescence staining of ICD markers in SCC7 allograft model under different treatment conditions. Representative confocal images showing calreticulin (CRT, green), HMGB1 (red), and DAPI-stained nuclei (blue) across six experimental groups (I–VI). Scale bar: 100 μm.


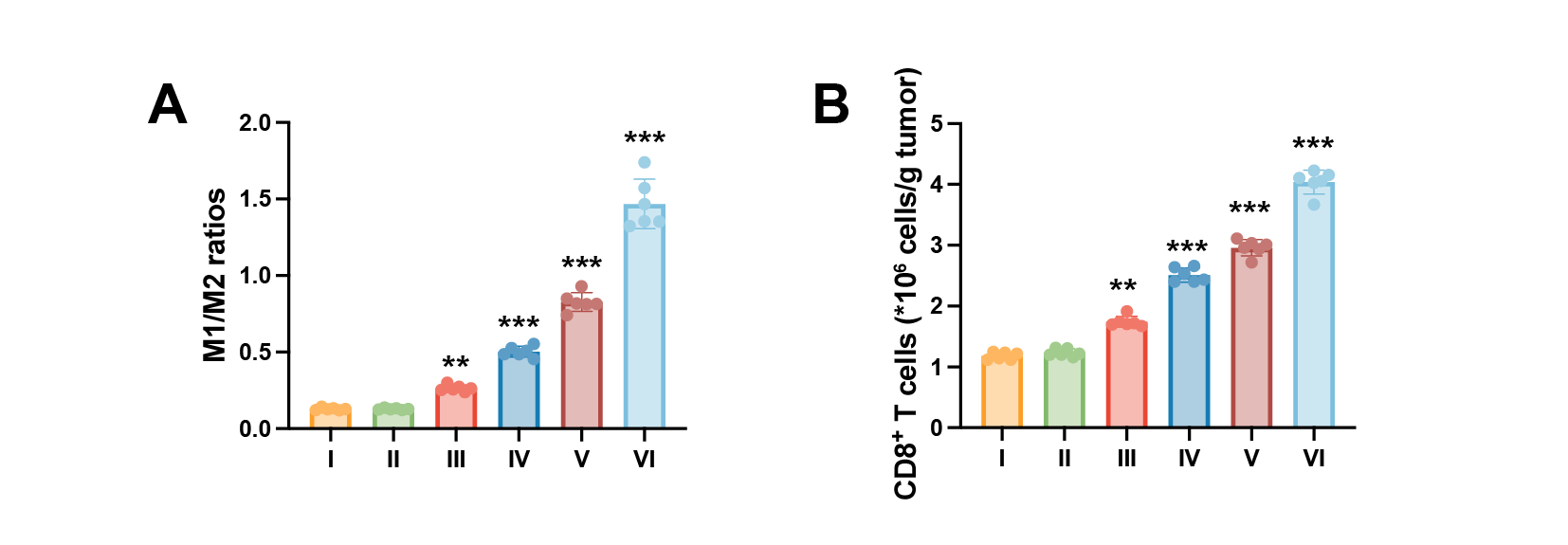


**Figure S18.** A) M1/M2 macrophage ratio in tumor tissues of each group (I–VI). B) CD8⁺ T cell infiltration per gram of tumor tissue (×10⁶ cells/g).


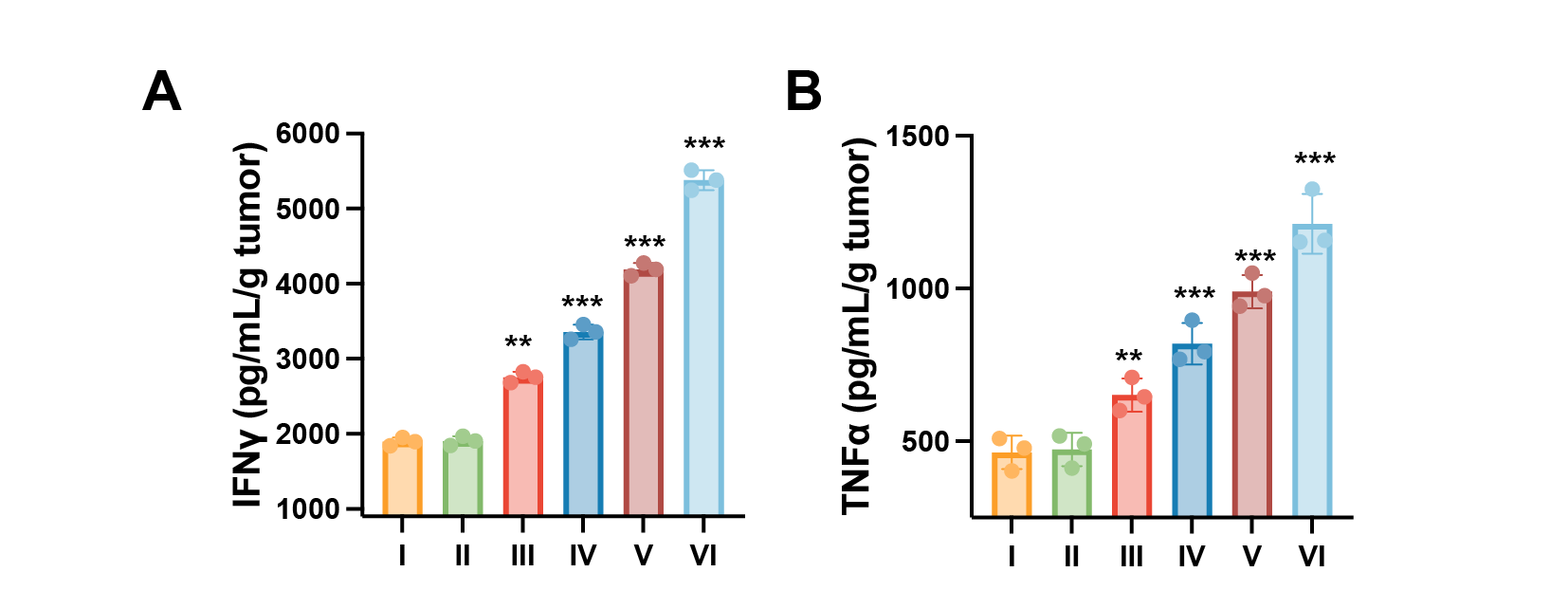


**Figure S19.** Intratumoral levels of IFN-γ (A) and TNF-α (B) in SCC7 tumor-bearing mice determined by ELISA following various treatments.


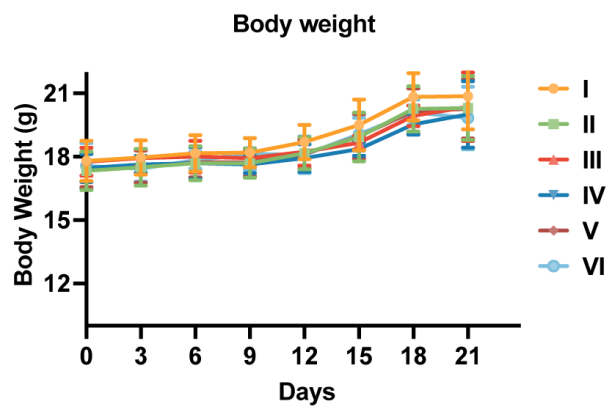


**Figure S20.** Changes in body weight during treatment. I: PBS; II: M_FC_; III: M_FC_ + 660 nm; IV: M@M_FC_ + 660 nm; V: BM@M_FC_ + 660 nm; V: BM@M_FC_C + 660 nm.


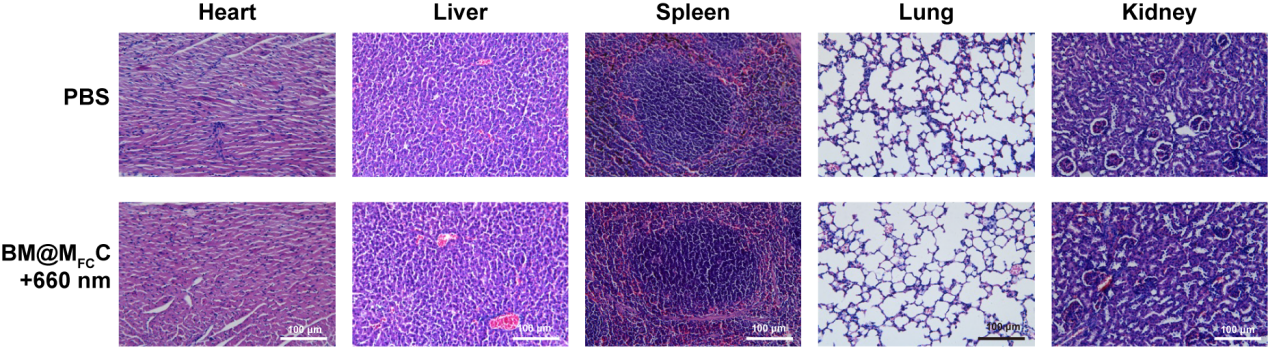


**Figure S21.** H&E staining of the heart, liver, spleen, and kidney tissues obtained from OSCC bearing mice models after treatments by PBS and BM@M_FC_C + 660 nm.


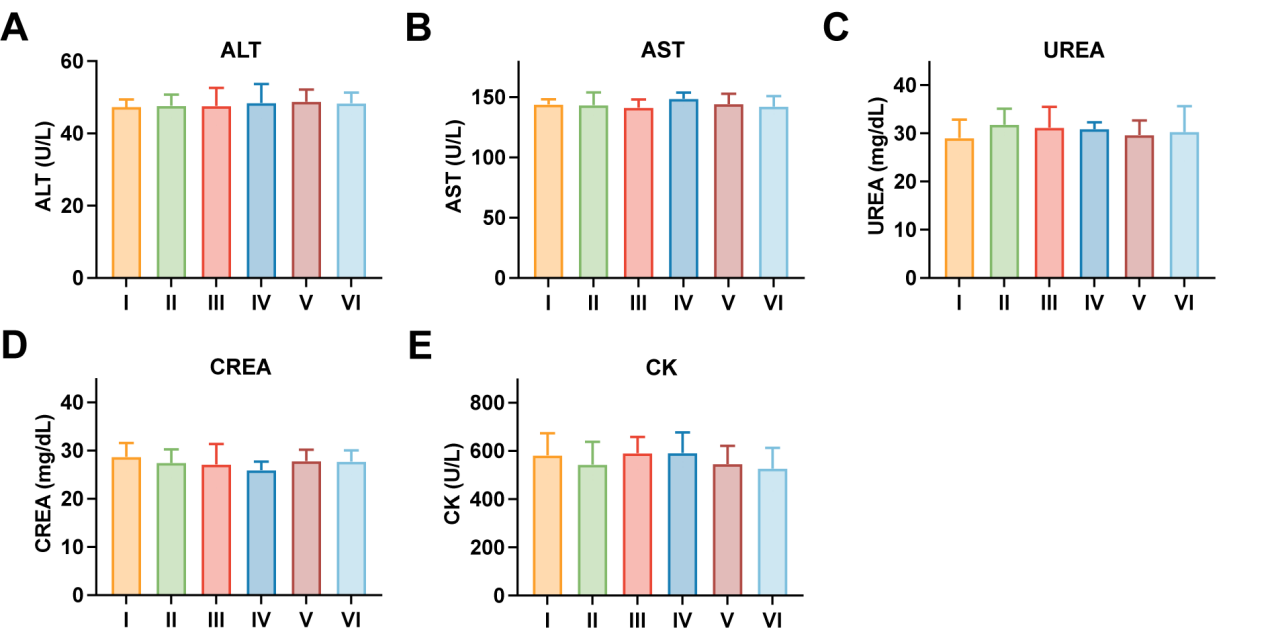


**Figure S22.** The serum biochemistry analysis of alanine transaminase (ALT), aspartate transaminase (AST), urea nitrogen (UREA), creatinine (CREA), or creatine kinase (CK) in mice after different treatments. I: PBS; II: M_FC_; III: M_FC_ + 660 nm; IV: M@M_FC_ + 660 nm; V: BM@M_FC_ + 660 nm; V: BM@M_FC_C + 660 nm.


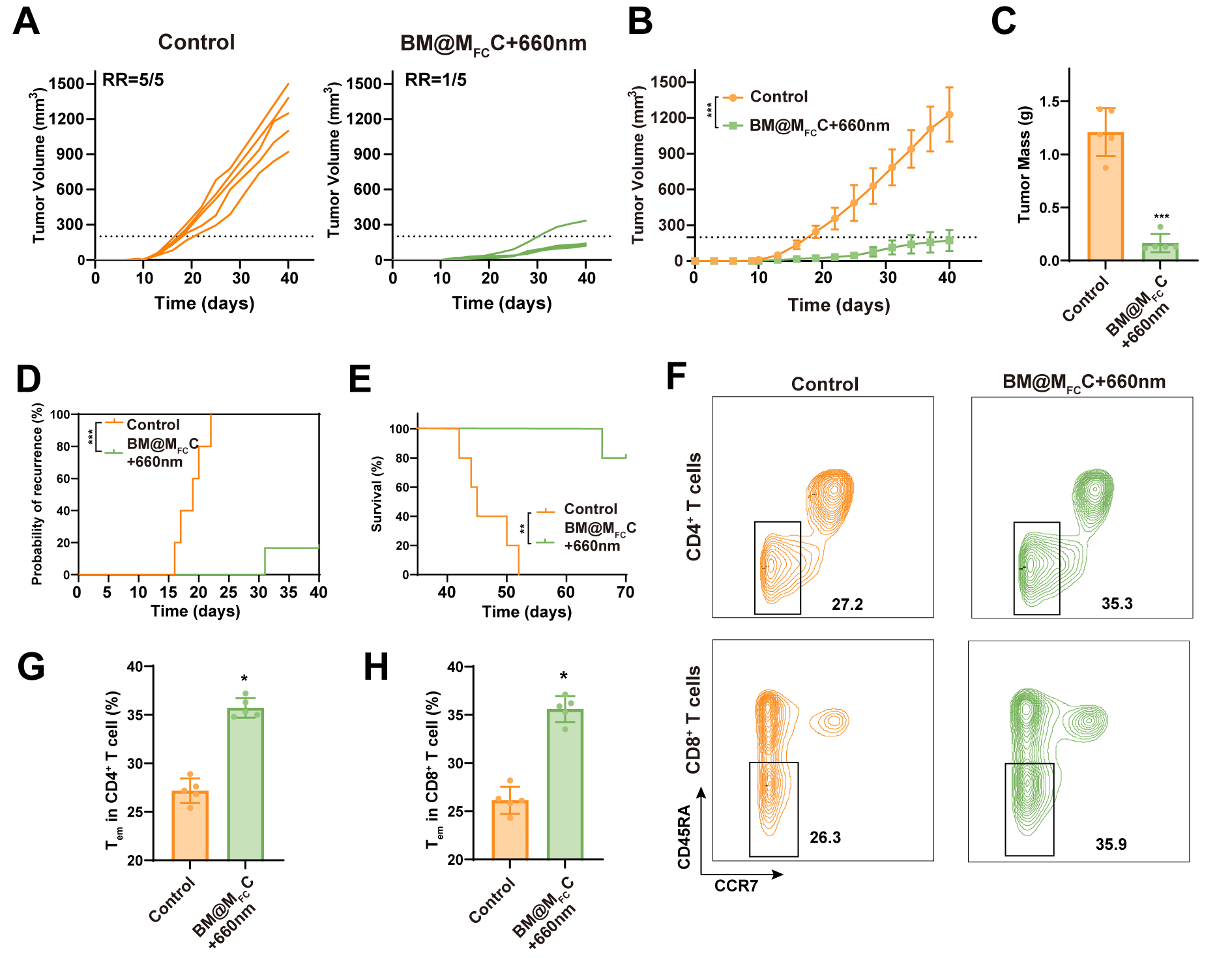


**Figure S23.** Antitumor efficacy and immune memory response in SCC7 tumor-bearing mice. A-C) Tumor growth inhibition and reduced tumor mass following BM@M_FC_C + 660 nm treatment. D, E) Tumor recurrence probability and overall survival analysis. F–H) Enhanced CD4+ and CD8+ effector memory T cell populations in cured mice.


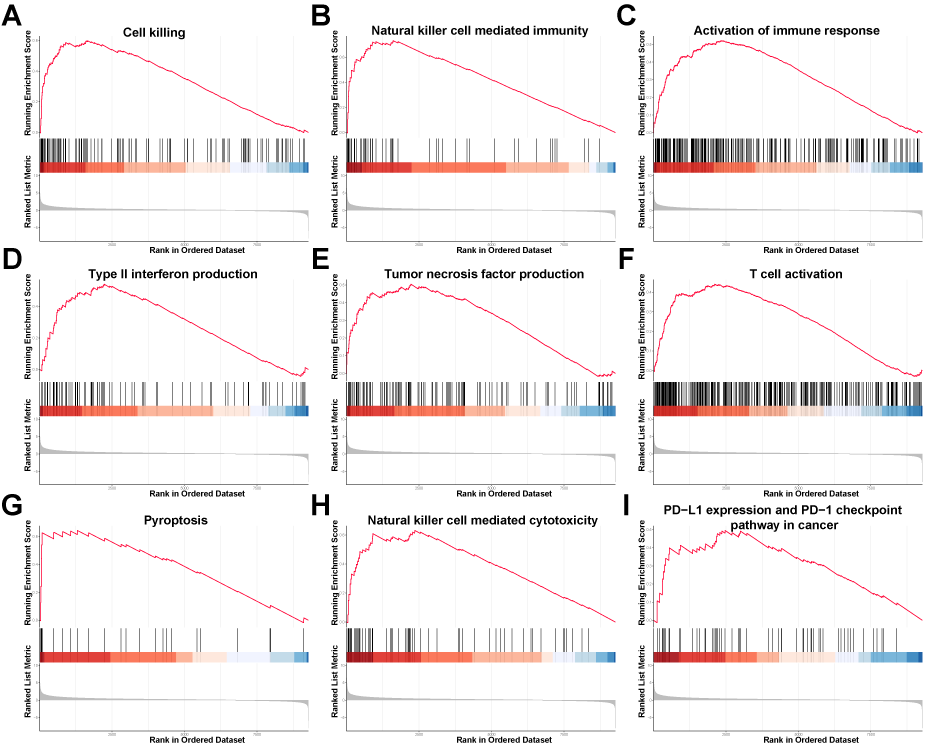


**Figure S24.** Gene Set Enrichment Analysis (GSEA) results of tumor malignant epithelial cells treated with or without BM@M_FC_C-mediated phototherapy.


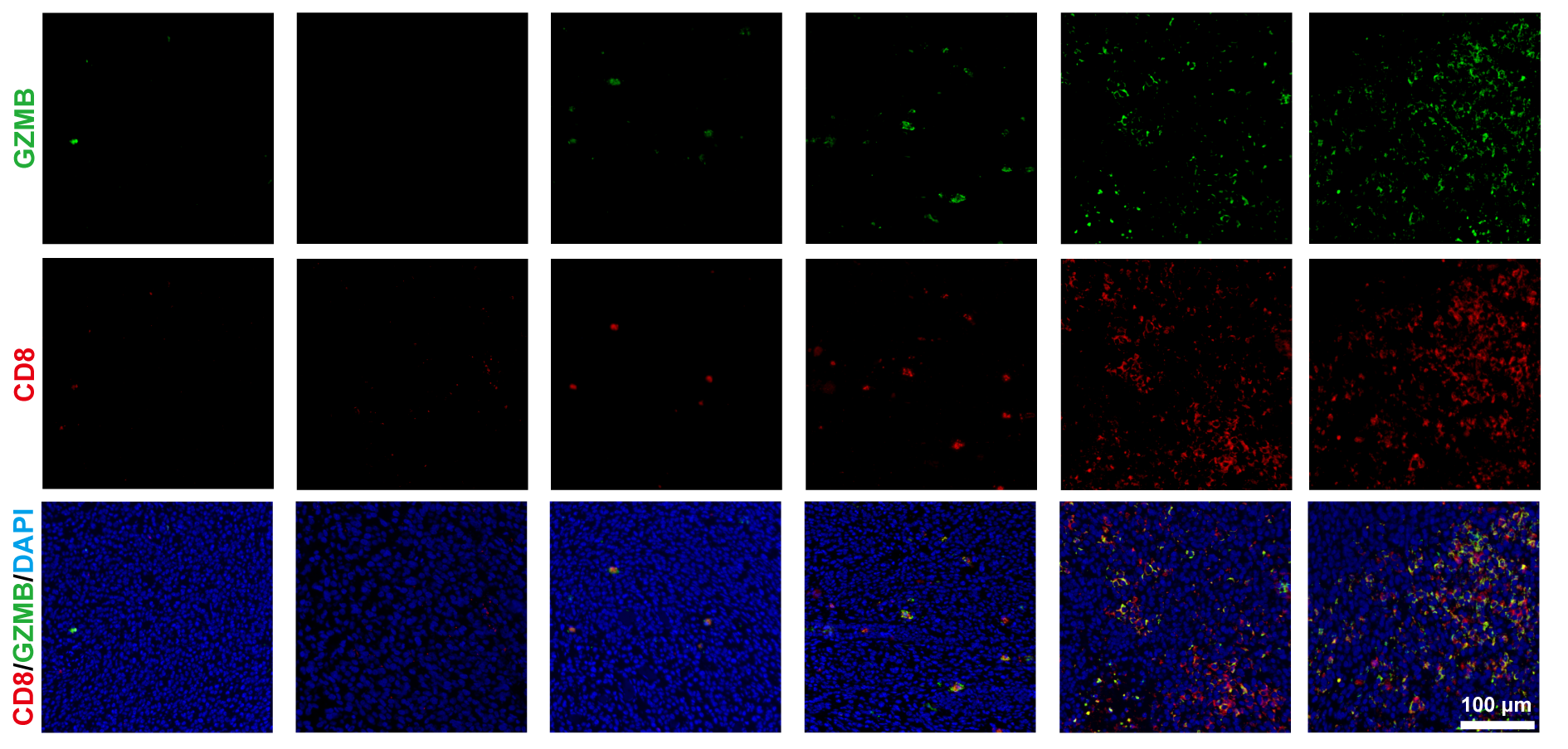


**Figure S25.** Fluorescent micrographs of CD8^+^ and GZMB^+^ T cells in the tumor tissues after different treatments.


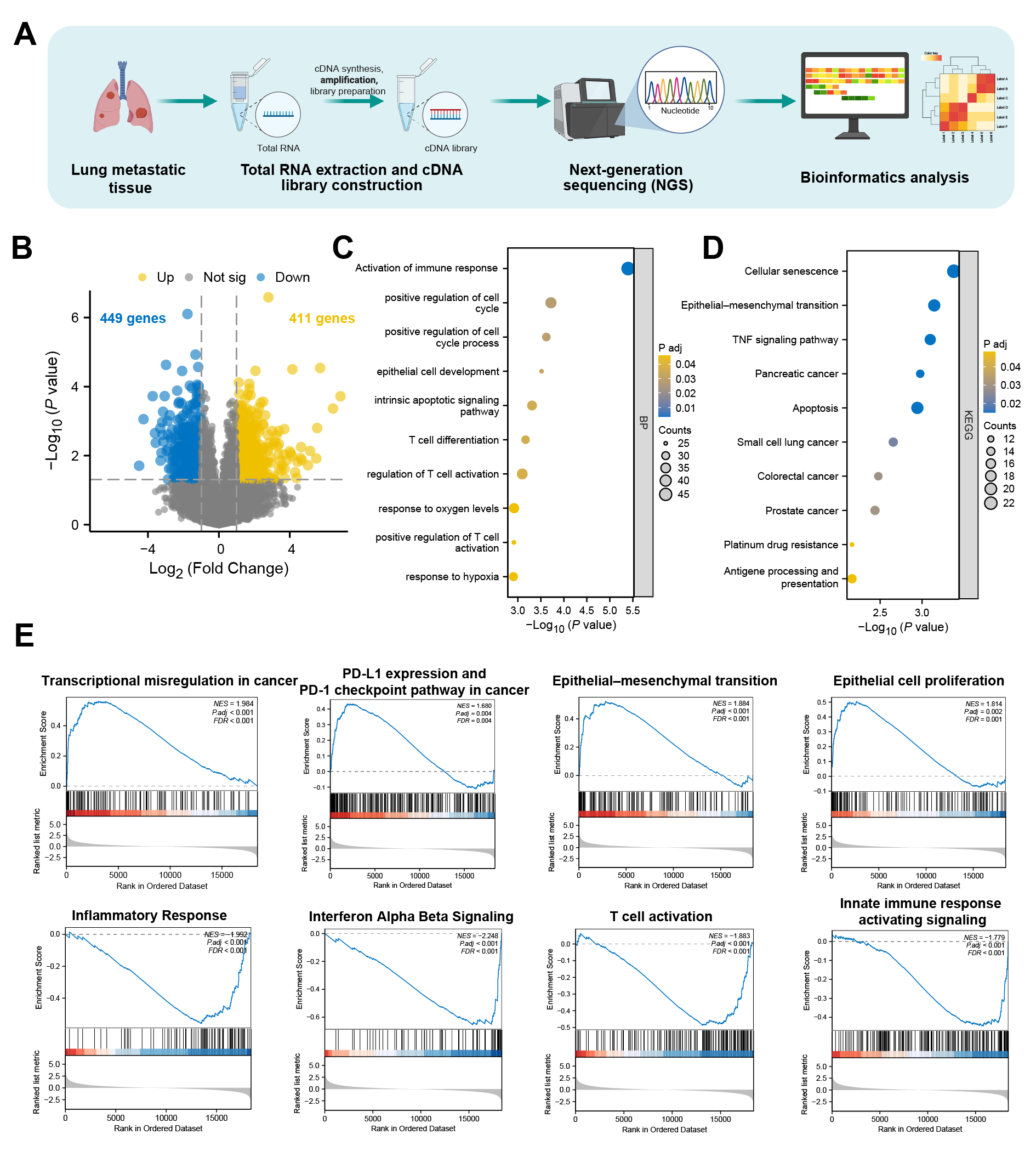


**Figure S26.** A) Schematic workflow of RNA sequencing. B) Volcano plot showing differentially expressed genes (DEGs) between the BM@M_FC_C+660 nm and control groups (fold change >1.5, P < 0.05); 449 upregulated (red) and 411 downregulated (blue) genes. C) GO enrichment analysis of upregulated genes, highlighting immune response, T cell activation, apoptosis, and hypoxia. (D) KEGG pathway enrichment analysis of upregulated genes, including antigen processing and presentation, PD-L1/PD-1 checkpoint, apoptosis, and EMT. E) GSEA showing enrichment of the transcriptional misregulation in cancer, PD-L1/PD-1 checkpoint pathway in cancer, EMT, epithelial cell proliferation, and negative enrichment of inflammatory response and interferon alpha/beta signaling, T cell activation, and innate immune response activating signaling in the control group relative to the treatment group. NES and adjusted P values are shown.
